# Research advances and new challenges in overcoming triple-negative breast cancer

**DOI:** 10.20517/cdr.2021.04

**Published:** 2021-04-08

**Authors:** Yu Zong, Mark Pegram

**Affiliations:** Stanford Cancer Institute, Stanford University School of Medicine, Stanford, CA 94305, USA.

**Keywords:** Triple negative breast cancer, subtypes, genomic evolution, therapeutic targets, homologous recombination deficiency, antibody-drug conjugates, immunotherapy, tumor immune microenvironment

## Abstract

Triple-negative breast cancer (TNBC) is a pathological term used to identify invasive breast cancers that lack expression of estrogen and progesterone receptors and do not have pathologic overexpression of the HER2 receptor or harbor *ERBB2* gene amplification. TNBC includes a collection of multiple distinct disease entities based upon genomic, transcriptomic and phenotypic characterization. Despite improved clinical outcomes with the development of novel therapeutics, TNBC still yields the worst prognosis among all clinical subtypes of breast cancer. We will systematically review evidence of the genomic evolution of TNBC, as well as potential mechanisms of disease progression and treatment resistance, defined in part by advances in next-generation DNA sequencing technology (including single cell sequencing), providing a new perspective on treatment strategies, and promise to reveal new potential therapeutic targets. Moreover, we review novel therapies aimed at homologous recombination deficiency, PI3 kinase/AKT/PTEN pathway activation, androgen receptor blockade, immune checkpoint inhibition, as well as antibody-drug conjugates engaging novel cell surface targets, including recent progress in pre-clinical and clinical studies which further validate the role of targeted therapies in TNBC. Despite major advances in treatment for TNBC, including FDA approval of 2 PARP inhibitors for metastatic TNBC, the crossing of the superiority boundary in a phase 3, placebo-controlled study of adjuvant olaparib in early-stage patients with germline BRCA-mutated high-risk HER2-negative early breast cancer, the FDA approval of 2 PD-(L)1 checkpoint antibodies for metastatic TNBC, and the FDA approval of the first antibody drug conjugate for TNBC, significant challenges remain. For example, despite the dawn of immunotherapy in metastatic TNBC, durable responses are limited to a small subset of patients, definitive biomarkers for patient selection are lacking, and the Oncology Drug Advisory Committee to the FDA has recently voted against approval of an anti-PD-1 checkpoint antibody high risk early-stage TNBC in the neoadjuvant setting. Also, despite early positive randomized phase 2 studies of AKT inhibition in metastatic TNBC, a recent phase 3 registration trial failed to validate earlier phase 2 data. Finally, we note that level one evidence for clinical efficacy of androgen receptor blockade in TNBC is still lacking. To meet these and other challenges, we will catalogue the ongoing exponential increase in interest in basic, translational, and clinical research to develop new treatment paradigms for TNBC.

## INTRODUCTION

Triple-negative breast cancer (TNBC) is a pathological term used to identify invasive breast cancers that lack the expression of estrogen and progesterone receptors (ER/PR) and do not harbor pathologic overexpression of the human epidermal growth factor receptor 2 (HER2) or amplification of the *ERBB2* gene. TNBC, accounting for approximately 15% of all invasive breast cancers^[1]^, is a rising burden globally^[2,3]^; although the cutoffs used to define “positive” *vs.* “negative” expression of steroid hormone receptors and *ERBB2* amplification/overexpression have changed over time^[4,5]^. Clinically, patients with TNBC are more likely to have higher grade tumors, earlier age of disease onset, and worse prognosis in terms of disease-free survival (DFS) and overall survival (OS)^[6–9]^. Moreover, TNBC shows a remarkable diversity of prognosis and clinical response to cancer treatment. A majority of the metastasis from TNBC occurs within the first three years following diagnosis^[10]^, but patients who have not recurred during this time have similar survival rates as patients with ER-positive breast cancers. Numerous historical neoadjuvant systemic treatment trials have shown that approximately 33% of TNBC patients achieve a pathological complete response (pCR) following neoadjuvant chemotherapy^[11]^. Indeed, even higher rates of pCR have been reported for patients with TNBC treated with platinum-based neoadjuvant chemotherapy regimens, 53.2% and 54% for the GeparSixto (NCT01426880), and CALGB 40603 (NCT00861705) clinical trials, respectively^[12,13]^. TNBC patients who experienced pCR at the time of surgery have significantly improved long-term outcomes compared to patients with residual invasive disease^[11]^, and have similar prognosis to those with non-TNBC^[8]^. However, for TNBC patients with residual disease after neoadjuvant chemotherapy, significantly worse survival and higher rates of relapse within the first three years after treatment are observed^[7]^.

Using cDNA microarray analysis for gene expression profiling (GEP), Perou *et al*.^[1]^ unveiled a distinctive “molecular portrait” of breast cancer representing five intrinsic subtypes with distinct clinical outcomes, i.e., luminal A, luminal B, HER2 overexpression, basal and normal-like tumors. Subsequently, Herschkowitz *et al*.^[14]^ identified a new intrinsic subtype, termed “Claudin-low”, which is characterized by the low to absent expression of luminal differentiation markers, high enrichment for epithelial-to-mesenchymal transition (EMT) markers, immune response genes and cancer stem cell-like features^[15]^. Clinically, the majority of claudin-low tumors are TNBC with a high frequency of metaplastic and medullary differentiation and an intermediate response rate to standard neoadjuvant chemotherapy, between that of basal-like and luminal tumors. When molecular classification based upon GEP was applied to TNBC, Prat *et al*.^[16]^ were able to identify a group of clinically “mixed” tumor phenotypes: 49% of TNBC were basal-like, 30% claudin-low, 9% HER2-enriched, 6% luminal B, 5% luminal A and 1% normal breast-like. These results shed light upon the complex heterogeneity of TNBC at the molecular level, and has inspired further research into subtyping of breast cancers which are clinically defined as TNBC^[16]^.

## TRIPLE-NEGATIVE BREAST CANCER SUBTYPING

Using gene expression analyses from 386 tumors, Lehmann *et al*.^[17]^ originally identified 6 distinct TNBC subtypes, each displaying unique biology. The TNBC molecular subtypes consist of 2 basal-like (BL1 and BL2), an immunomodulatory (IM), a mesenchymal (M), a mesenchymal stem-like (MSL), and a luminal androgen receptor (LAR) subtype [Table 1]. The BL1 subtype has features of highly activated cell division pathway components and DNA damage response (ATR/BRCA) pathways. The BL2 subtype is enriched for growth factor signaling (EGF, NGF, MET, Wnt/β-catenin and IGF1R pathways), glycolysis and gluconeogenesis, and the expression of myoepithelial markers (TP63 and MME). Gene ontologies of the IM subtype are mainly focused on immune cell processes and immune signal transduction pathways. The M and MSL subtypes are enriched in motility, extracellular matrix interactions, cell differentiation pathways and genes associated with EMT. A unique feature of the MSL subtype which distinguishes it from the M subtype is that it expresses low levels of proliferation genes and claudin 3, 4, 7 and has elevated expression for mesenchymal stem cell-associated genes. Interestingly, the LAR subtype displays an enrichment of hormonally regulated pathways such as steroid synthesis, porphyrin metabolism, and androgen/estrogen metabolism, even though TNBC is defined as ER/PR negative. Genes including AR and its downstream signaling targets/co-activators are highly expressed as well. Moreover, Lehmann *et al*.^[17]^ categorized commonly-used TNBC cell lines into different representative subtypes by GEP and targeted predicted “driver” signaling pathways pharmacologically *in vitro* as a proof of concept that molecular classification of TNBC may be exploited clinically. Indeed, a retrospective clinical study showed TNBC subtype was an independent predictor of pCR status (*P* = 0.022) - the BL1 subtype had the highest pCR rate (52%); BL2 and AR had the lowest (0% and 10%, respectively)^[18]^. It is hypothesized that granularity in classification of TNBC explains clinical heterogeneity of response and prognosis, and provides insights into novel treatment paradigms informed by molecular analysis.

In 2016, Lehmann *et al*.^[19]^ refined the TNBC molecular subtypes from 6 into 4 (TNBCtype-4) tumor-specific subtypes (BL1, BL2, M and LAR) after taking into consideration that IM and MSL subtypes were primarily impacted by contamination from normal stromal and immune cells in the tumor environment instead of tumor cells *per se*. The 4 TNBC subtypes demonstrated distinguishable clinicopathological features, including age at diagnosis, histopathology, tumor grade, and disease progression. However, TNBCtype-4 subtyping did not predict pCR for neoadjuvant chemotherapy in TNBC patients. The latter classification was also supported by a similar transcriptional analysis of 198 previously uncharacterized TNBCs using mRNA expression and DNA profiling, which identified 4 stable TNBC subtypes with distinct clinical prognosis: luminal AR (LAR), mesenchymal (MES), basal-like immunosuppressed (BLIS), and basal-like immune-activated (BLIA)^[20]^. Between the 2 studies, there is evident overlap between MSL and MES, IM and BL1 with BLIA, M with BLIS, and the 2 LAR subtypes. The prognosis analysis showed that disease-free survival (DFS) was in the order of BLIA > M > LAR > BLIS (*P* = 0.019) and disease-specific survival (DSS) showed the order of BLIA > M > LAR > BLIS (*P* = 0.07). We can conclude from the aforementioned studies that transcriptional profiling is a reliable and reproducible method to subtype TNBC, and that subtype-specific somatic alterations have been employed as treatment targets for preclinical and clinical drug development, which may enlighten future treatment paradigms.

## UNDERSTANDING THE DYNAMICS OF TRIPLE-NEGATIVE BREAST CANCER DISEASE PROGRESSION

Tumor progression is now recognized as an evolutionary process which provides a foundation for studying the dynamics of tumor growth and resistance. The revolution in next-generation sequencing technology has provided a wealth of new data uncovering the immense genomic complexity of cancer evolution^[21]^. To decipher the narratives of TNBC encrypted in the genetic alterations, we must not only focus on a single time point (for example, the breast cancer genome within the primary tumor site at the time of diagnosis), but also deconstruct underlying clonal dynamics and measure changes in tumor composition across time (for example, primary *vs.* metastatic) and space (both intra-tumoral spatial heterogeneity, as well as geographic variation in genomic profiles in different metastatic sites) via proper mathematical models. Understanding the complexity of TNBC clonal dynamics imposed by intrinsic evolutional pressure and by selection pressure from various systemic therapies will help us identify, and hopefully exploit the drug resistance mechanisms that evolve through time and anatomical (including intratumoral) location.

In 2012, Shah *et al*.^[22]^ described a broad and continuous spectrum of mutational content and clonal composition in primary TNBC clinical samples. Genome aberrations at all scales from 104 primary TNBC patients were enumerated by single-nucleotide polymorphism (SNP) array, RNA sequencing (RNA-seq) and genome/exome sequencing. As expected, the distribution of somatic mutations and copy number aberrations (CNAs) varied among patients, but different clonal frequencies of mutations were observed within individual tumors, indicating distinct clonal genotypes.

The advent of single-cell sequencing has enabled researchers to explore genomic diversity at single-cell resolution. Wang *et al*.^[23]^ performed population (bulk) sequencing of tumor (72x) and matched normal tissue (74x) in one treatment-naive primary TNBC case, followed by whole-genome and exome sequencing of 16 single tumor nuclei from the G2/M peaks and 16 single normal nuclei. Hierarchical clustering and multi-dimensional scaling were based on nonsynonymous point mutations. Three classes of mutations were identified in this study: (1) clonal mutations, detected in the bulk tumor sample and most single tumor cells; (2) subclonal mutations, identified in more than 2 single cells, but not in the bulk tumor; and (3) *de novo* mutations, recognized only in one tumor cell (which were difficult to distinguish from technical errors). Hence, targeted deep-sequencing was then performed to verify the mutations identified by single-cell sequencing and assess the mutational frequencies in the bulk tumor. Interestingly, the results demonstrated that no 2 single tumor cells are genetically identical. In all, 374 clonal nonsynonymous mutations identified by bulk sequencing were also detected in most single tumor cells at high frequencies. Moreover, single-cell sequencing also identified 145 additional subclonal mutations and *de novo* mutations not seen by bulk sequencing. These findings proved that despite being less prevalent (even rare), these mutations were real biological variants occurring at low frequencies (instead of technical misinterpretation) and may play a central part in broadening the phenotypes of TNBC, and allow them to survive intrinsic selective pressures. According to the mathematical model developed with single-cell mutation frequencies, the TNBC cells produced about 8 mutations per cell division, 13 times more than normal breast cells, which enabled the accumulation of a large number of diverse mutations and contributed to the emergence of major clones and tumor evolution in the primary TNBC even before the introduction of any therapy^[23]^.

Thus, there were 2 opposite theories regarding the formation of clonal diversification involving mutations. Yates *et al*.^[24]^ applied multi-region sequencing to 303 breast cancer samples and no strict temporal order was perceived in subclonal diversification across breast cancer subtypes, suggesting commonly seen point mutations (clonal mutations) such as *PIK3CA*, *TP53*, *PTEN*, *BRCA2* (somatic) and *MYC* may take place any time in tumor initiation and progression. However, it was not consistent with the fundamental sequencing work of TNBC by Shah *et al*.^[22]^, in which clonality analysis demonstrated that known driver mutations such as *TP53*, *PIK3CA* and *PTEN* possessed the highest clonal frequencies (suggesting that they were early events in tumorigenesis), whereas mutations involved in cell shape/motility and extracellular matrix-signaling pathways occur at lower clonal frequencies and later on the cancer evolution time scale.

In contrast to the diversity of point mutations in TNBC, copy number profiles were shown to be surprisingly similar, raising the question how CNAs were acquired in TNBC tumor evolution. Gao *et al*.^[25]^ explored the copy number evolution in treatment-naive TNBC using a highly multiplexed single-nucleus sequencing method. One thousand single cells from 12 TNBC cases were sequenced, and 1–3 major clonal subpopulations in each tumor that shared a common evolutionary lineage were identified. The single cells had highly conservative CNA profiles within each subpopulation, representing stable clonal expansions (clonal stasis) during tumor growth. Meanwhile, it was also seen that subclonal CNAs were related to increased genotype frequencies of the individual clones, indicating that TNBC can continue to acquire CNAs during tumor progression in addition to stable clonal expansions, causing the increased prevalence of emerging subpopulations. Phylogenetic tree construction and mathematical modeling supported that in TNBC, most CNAs were acquired at the earliest phases of tumor evolution in short bursts of crisis, followed by stable clonal expansions with tumor outgrowth. The punctuated copy number evolution hypothesis challenges the conceptual foundation of gradual evolution for tumor growth, and supports the “Big Bang” model in which clonal diversification accumulated at the earliest phases (i.e., subclinical) of tumor formation, followed by stable expansion of specific clones^[26,27]^.

Extensive studies of primary breast cancer have provided clear evidence of clonal evolution and have helped to identify a collection of pathogenic driver gene mutations and passenger events. Clinically, metastases derived from primary cancer are the leading cause of mortality, instead of the primary breast cancer itself. Hence, in-depth genomic analysis focusing on TNBC metastases could decipher the active molecular processes in the more deadly form of the disease. *De novo* stage IV breast cancer provides a valuable testing ground to explore differences in the genetic alterations without the selective pressures imposed by systemic therapies. Any alteration observed would be an intrinsic property of the cancer and not due to treatment effects. Ng *et al*.^[28]^ collected synchronous primary and metastasis samples from 9 *de novo* stage IV, treat-naive breast cancer patients, including 2 TNBC cases. Somatic mutations and CNA profile data were characterized by whole-exome sequencing with an average depth of 200x. After comparing the somatic mutations and copy number profiles in the paired primary and synchronous metastatic lesions, a median of 60% (ranging from 6% to 95%) of somatic mutations, 62% (29/47) of focal amplifications and homozygous deletions were detected in both primary and metastatic samples. The amount of somatic mutations was significantly increased in the metastatic lesions when compared to the primary tumors. The most commonly mutated genes in breast cancer (considered as driver mutations) including *TP53*, *PIK3CA*, and *GATA3* were detected in both the primary and metastatic lesions, most of which were indeed clonal in both lesions. The heterogeneity observed between primary and metastatic lesions was more prominent in subclonal (“passenger”) genetic alterations. For instance, mutations involving EMT-related genes, such as *SMAD4*, *TCF7L2*, and *TCF4* (*ITF2*), were restricted to the metastatic lesions. To note, the emerging subclonal genetic aberrations (mutations and/or CNAs) were irrespective of breast cancer subtype. Therefore, this study supported the hypothesis that an evident temporal order should be expected in the TNBC clonal evolution when driver mutations emerged before the tumor metastasis and were passed on with clonal expansion and domination. With the continuous accumulation model of somatic mutations during disease progression, synchronous breast cancer metastasis had a rather different repertoire of somatic genetic alterations from its primary lesions^[28]^.

More recently, Bertucci *et al*.^[29]^ conducted a study with a much larger sample size, comparing the frequency of alterations integrated with somatic mutations, CNAs, mutational signatures, and tumor mutational burden (TMB) in metastatic breast cancer to early breast cancer using data from the Cancer Genome Atlas (TCGA). Whole exome sequencing was performed in 617 metastatic breast cancers, 182 of which were TNBC. Even though for 9 common cancer driver genes (*TP53, ESR1, GATA3, KMT2C, NCOR1, AKT1, NF1*, *RIC8A* and *RB1*), no further enrichment was observed in metastatic TNBC when compared to early TNBC, and increased TMB and clonal diversity were observed in metastatic TNBC. In addition, a whole-genome sequencing study involving 442 metastatic breast cancer patients reported by Angus *et al*.^[30]^ concurred that mutation frequency of 21 potential driver genes was equivalent in metastatic TNBC and in early breast cancer cases from BASIS cohort^[31]^, whereas TMB was significantly higher in metastatic breast cancer irrespective of breast cancer subtypes. Hence, we can infer that activation of mutational processes may drive genome evolution from primary to metastatic breast cancer and contribute to the genetic complexity of the metastatic tumors, suggesting that therapeutics targeting early mutational events may need to be included in the treatment strategy as early as possible.

In addition to the intrinsic evolutional drive in TNBC, anti-cancer treatment serves as the other source of selection pressure. To this date, it still remains controversial whether chemotherapy resistance emerges from the selection and expansion of rare pre-existing subclones (adaptive resistance) or through the induction of new mutations (acquired resistance). Several studies have approached this conundrum using neoadjuvant chemotherapy (NAC) as an ideal testing ground. Early evidence was provided by a study employing targeted sequencing of oncogenes, tumor suppressor gene exons and frequently rearranged introns in 20 pairs of pre- and post-NAC residual TNBC samples^[32]^. After adjustment for regional sampling/tumor purity bias between the matched specimens, targeted sequencing data did not identify any significant changes in genomic alterations as a result of NAC, suggesting most alterations may not be selected for or against by chemotherapy. Another early study employing *in situ* hybridization methods also described consistent genetic diversity pre- and post-NAC in a cohort of 47 primary breast cancer patients (12 TNBC) at a single-cell basis^[33]^. Immunofluorescence *in situ* hybridization (iFISH) was subjected to detect gain/loss of chromosomal regions with probes for genomic loci 8q24.3, 10p13, 16p13.3, and 20q13.31 and the corresponding centromeric probes. Consistent with the previous study, no significant differences regarding amplification of selected chromosomal regions were detected in any pre- and post-NAC paired samples, while phenotypic shift illustrated by the decreased proportion of CD44^+^CD24^-^ cells, which possess tumorigenic, stem cell-like features^[34]^ and features of EMT^[35]^, was observed post-NAC by immunofluorescence (IF) staining. Both studies suggested that the genetic alterations were persistent despite cytotoxic treatment.

A limitation of the above-mentioned studies is evident in that both studies were based on targeted markers and lack the ability to reconstruct clonal evolution during and after chemotherapy. Using whole-genome sequencing, Yates *et al*.^[24]^ sequenced 18 breast cancers with both diagnostic biopsies and residual invasive disease specimens following NAC (with the mean depth of 166x). In 6 cases, subclonal mutations were detected in both samples prior to and following NAC, whereas in 5 cancers, one subclone was only identified in the residual tumor after NAC. Detailed phylogenies generated in 3 pairs of pre- and post-NAC samples suggested both subclones pre- and post-NAC had a similar “molecular age”. Further mutational signature profiles also suggested that chemotherapy-induced mutagenesis had a minimal contribution to genetic heterogeneity observed in pre- and post-NAC breast cancer. Therefore, the authors inferred that subclonal mutations detected only in post-NAC samples were rare pre-existing subclones in the primary breast cancer but not captured in the pre-NAC biopsy most likely due to spatial heterogeneity^[24]^.

Kim *et al*.^[36]^ used single-cell DNA whole-exome sequencing and RNA-seq to enable phylogenetic reconstruction of tumor lineages in 20 primary TNBC patients treated with anthracycline and taxane-based NAC. An exciting insight provided by this work is that compared to previous studies only working with residual TNBC, the authors included 2 distinct groups of patients: clonal extinction *vs.* clonal persistence patients defined by whether somatic mutations (nonsynonymous mutations and indels) were detectable following NAC. This classification was also supported by the analysis of ploidy - for clonal extinction patients, aneuploidy clusters were found exclusively in the pre-NAC samples, whereas the diploid clusters were more likely to be detected in the post-NAC samples. This classification mirrored 2 clinical outcomes of TNBC patients after NAC, those who achieved pCR and those with residual disease. In clonal persistence patients, the vast majority of mutations identified in post-NAC samples indeed existed in the pre-NAC tumor, but at low frequencies, they were adaptively selected. Most subclones shared CNAs and shared common evolutionary ancestors, indicating CNAs detected after NAC were also pre-existing. The pre-existence of the chemo-resistant genetic aberrations again supported the hypothesis of adaptive resistance in TNBC. Interestingly, it was also observed that a small fraction of phenotypic features by transcriptional profiles were only existent in the post-treatment samples, which could not simply be explained by adaptive resistance theory.

Collectively, TNBC displays a complex spectrum of genetic heterogeneity and dynamic clonal evolution. Current evidence supports that the formation of TNBC clonal diversification happens early in the tumor initiation and progression, probably driven by increased mutation rate, with ongoing mutational shaping in the subclones. Clonality analysis demonstrated that while common driver mutations could be early events in tumor evolution (even founder events), other rarer mutations may play an important role in shaping phenotypes of cancer cells, enabling them to survive selection pressure in tumor microenvironments. Moreover, the pre-existence of chemo-resistant genomic alterations identified before the evolutional challenges imposed by anti-cancer therapies not only answers the question of how drug resistance is derived, but may also emphasize the importance of early detection of clonal alterations prior to treatment administration, some of which may represent ideal therapeutic targets to prevent the emergence of drug resistance.

## CONSIDERATION OF KEY NOVEL THERAPEUTIC TARGETS CURRENTLY BEING EXPLORED IN TRIPLE-NEGATIVE BREAST CANCER

### BRCA1/2 mutation and homologous recombination deficiency

The prevalence of a germline *BRCA* mutation ranges from 1.2 to 8.8% in unselected breast cancer patient populations^[37]^ and 15% in unselected patients with TNBC^[38]^. *BRCA1* and *BRCA2* genes encode proteins critically involved in the pathway of DNA double-strand break repair by the process of homologous recombination (HR). Poly (ADP-ribose) polymerase (PARP) enzymes play an essential role in the maintenance of genomic stability for resolving stalled replication forks, detecting DNA double-strand breaks, and mediating the recruitment of additional DNA repair factors to damaged DNA lesions^[39]^. Targeting DNA damage response pathways may therefore be exploited clinically as an attractive strategy to destabilize tumor genomic integrity and trigger genomic catastrophe and cell death. Breast cancer patients with *BRCA1/2* germline mutations respond favorably to therapies that target DNA repair pathways, such as platinum salts and PARP inhibitors^[40–42]^. Indeed, the first positive randomized phase III clinical trial results for PARP inhibition in BRCA-mutant high risk early-stage breast cancer have just been announced^[43]^. Moreover, it is recognized that some sporadic TNBC biologically resembles breast cancers harboring germline *BRCA* mutations and may show comparable sensitivity to DNA-damaging agents^[44]^. It has been estimated that up to 40% of familial and sporadic breast cancers are HR deficient^[45]^. Hence, different approaches are being investigated to identify *BRCA1/2* wild-type tumors that can benefit from DNA-damaging agents and PARP inhibitors based on the presence of homologous recombination deficiency (HRD), including genomic alteration such as mutations and CNAs^[31,46]^, genome instability^[47–49]^, mutational signatures^[31,50]^ and epigenetic modification^[51–53]^.

One of the most widely investigated assays is the HRD score, defined by a combination of 3 DNA-based metrics of structural rearrangements including telomeric allelic imbalance (TAI), large-scale transition (LST), and loss of heterozygosity (LOH) detected by SNP profiling^[47–49]^, also known as “genomic scars”. A numeric sum of 3 scores was found to be more efficient in identifying *BRCA1/2* defects than individual scores^[54]^. However, cutoffs used to define HRD scores are not consistent among all of the published clinical trial datasets - an HRD score ≥ 42 was used in early retrospective studies, including the GeparSixto trial (NCT01426880) and the TNT trial (NCT00532727). In contrast, an HRD score ≥ 33 was used in the TBCRC 030 trial (NCT01982448)^[55]^ after improved sensitivity to predict responders to additional carboplatin and PARP inhibitors was observed in BrighTNess trial (NCT02032277)^[56]^. When defined as HRD score greater than defined cutoffs and/or presence of tumor *BRCA* mutations, HRD was detected in about 40%−70% TNBC patients^[39,42,57–59]^. About 60% of patients with high HRD scores did not carry tumor BRCA mutations. Currently, the predictive value of HRD score for pathological response after NAC is still controversial. Both the GeparSixto trial (paclitaxel and non-pegylated liposomal doxorubicin ± carboplatin, pCR 50% *vs.* 24.6%, *P* < 0.001) and BrighTNess trial (paclitaxel ± carboplatin/veliparib followed by doxorubicin and cyclophosphamide, higher pCR across all treatment groups) supported that patients with HRD had higher pCR rates compared to non-HRD patients, whereas in TBCRC 030 trial HRD score was not predictive of pathologic response (cisplatin or paclitaxel, RCB-0/1)^[55]^. Cumulative evidence from all current clinical trials does not yet support using HRD score routinely in the clinic as a predictor for platinum response. Indeed, in contrast to data from early-stage breast cancer, in the TNT trial HRD score in the metastatic setting failed to demonstrate its predictive value for response to platinum-based chemotherapy^[42]^. Notably, archival tissue blocks from the primary tumors were used for HRD score detection in the TNT trial, and thus it has been suggested that such analysis from the primary tumor may not have necessarily reflected the status of the HR pathway in the metastatic tumor compartment.

More recently, a different approach has emerged to define HRD by utilizing mutational signature analysis. Somatic mutational signatures (patterns of mutations and rearrangement) record DNA damage and DNA repair processes during tumorigenesis and reflect past/ongoing exposures to environmental insults (for example UV radiation), endogenous biochemical degradation, and DNA damage due to deficient HR pathways. A landmark study of 560 whole genomes revealed 12 base substitutions, 6 rearrangement signatures, and 3 signatures associated with defective HR-based DNA repair - one signature corresponded to absence of BRCA1 function, another correlated with BRCA2 deficiency, while another signature was related to *BRCA1* mutations/promoter hypermethylation and BRCA2 mutations^[31]^. Based on this theory, Davies *et al*.^[50]^ selected 6 distinct mutational signatures that predicted BRCA1/2 dysfunction including microhomology-mediated deletions, a base-substitution signature, rearrangement signatures, HRD index and a base-substitution signature, and integrated them into a weighted model, called HRDetect. An HRDetect score may be generated for each tumor based on the probability of BRCA1/2 deficiency with a probabilistic cutoff set at 0.7. When applied in a cohort of 560 breast cancer patients with 22 germline *BRCA1/2* mutation carriers as the positive control, HRDetect detected additional 33 tumors with a germline *BRCA1/2* mutation, 22 tumors with a somatic *BRCA1/2* mutation, and 47 samples with high HRDetect scores (> 0.7) without harboring *BRCA1/2* mutations, increasing the predicted BRCA1/2-deficiency rate to 22% (124/560) with the sensitivity of 98.7%. This new model was recently validated in a Swedish cohort of 254 primary TNBC patient samples^[60]^. Using defined cutoffs, 59% of patients were classified as HRDetect-high (> 0.7), of which 67% displayed germline/somatic mutations of *BRCA1/2, BRCA1* promoter hyper-methylation, *RAD51C* hyper-methylation or biallelic loss of function *PALB2*; 35.9% were identified as HRDetect-low (< 0.2) and 5.5% as HRD-intermediate (0.2–0.7). With HRDetect used as a reference, the previously described HRD score has a sensitivity of 87% and specificity of 84%, suggesting that mutational signature assessment might be more accurate in identifying HR deficient tumors that are responsive to platinum-based chemotherapy or PARP inhibition. Multivariable Cox regression analysis showed that in patients treated with adjuvant chemotherapy (fluorouracil, epirubicin and cyclophosphamide ± docetaxel), HRDetect classification was an independent prognostic factor with significantly improved invasive disease-free survival (HR = 0.42, 95%CI: 0.20–0.87) and distant relapse-free interval (HR = 0.31, 95%CI: 0.13–0.76) in HRDetect-high patients than in HRDetect-low patients, whether or not a genetic/epigenetic *BRCA1/2* aberration was identified. Moreover, HRDetect-low cancers were enriched for PI3 kinase/AKT1 pathway abnormalities, indicating potentially actionable targets for further treatment. Reassuringly, similar results were also reported by Chopra *et al*.^[61]^, employing 26 untreated primary TNBC samples from the RIO trial (EudraCT 2014–003319-12), a phase 2 clinical trial to identify predictive markers for PARP inhibitor response in TNBC. Seventy percent of patients were identified as HRDetect positive (> 0.7), the majority of which could be explained by inactivating mutations and promoter methylation of HR genes. The *ad hoc* analysis suggested that HRDetect was more specific to detect cancers with deficient HR than HRD score^[61]^. Other mutational signature assays such as Signature Multivariate Analysis (SigMA) have also been reported to accurately detect a mutational signature associated with HRD from targeted gene panels instead of whole-genome data. The predictive value of SigMA for platinum response has been determined in ovarian cancer patients^[62]^.

In addition to the significant efforts that have been made to accurately identify patient populations with “BRCAness” phenotypes that will benefit significantly from PARP inhibitors and platinum treatment, researchers are also trying to extend the indication of DNA damaging treatments by provoking BRCAness even in non-HRD cancers. Quereda *et al*.^[63]^ developed a selective dual CDK12/CDK13 inhibitor, SR-4835, which was reported to reduce the expression of core DNA damage response genes by increasing intronic polyadenylation site cleavage and result in provoking lethal accumulation of chemotherapy-induced DNA damage and augmenting the anticancer activity of cisplatin, irinotecan and olaparib even in an HR-competent TNBC mouse model. Such provocative treatment paradigms could be piloted in future early-phase clinical trials for further validation in translational fashion.

### Inhibition of the PI3K/AKT/mTOR pathway in triple-negative breast cancer

The PI3K/AKT signaling pathway plays an essential role in carcinogenesis by promoting cell survival and proliferation^[64]^. The large-scale comprehensive molecular landscape of breast cancer carried out by The Cancer Genome Atlas Network demonstrated a clear picture of PI3K/AKT/mTOR signaling pathway in TNBC, more precisely, in the basal-like breast cancer subtype. *PIK3CA* was the second most commonly mutated gene (9%) next to *TP53*^[65]^; and PI3K/AKT/mTOR pathway in basal-like cancers can be activated through *PIK3CA* or *AKT1* activating mutations and/or *PTEN* loss^[65,66]^. Moreover, targeted sequencing measurements of allelic abundance for 2414 somatic mutations and clonality analysis emphasized that frequent *PIK3CA* (10.2%) and *PTEN* (7.7%) somatic mutations seemed to be clonally dominant, consistent with their putative roles in early tumorigenesis^[22]^. This assumption was supported by the clinical observation that in one study the incidence of *PIK3CA*/*AKT1*/*PTEN* alterations was generally similar in primary breast cancer and TNBC metastasis samples^[67]^ (some temporal discordance between *PIK3CA* sequence in primary *vs.* metastasis in a small fraction of hormone receptor positive, HER2-negative breast cancers, notwithstanding^[68,69]^). Alternative means of activating the PI3K pathway in basal-like cancers probably include loss of *PTEN* and *INPP4B* and/or amplification of *PIK3CA*^[65]^. Molecular heterogeneity of PI3K pathway activation among molecular subtypes of TNBC has also been noted - BL1 subtype showed frequent *PIK3CA* amplification as well as *PIK3CA*, *AKT2* and *AKT3* overexpression; LAR tumors displayed significantly enriched *PI3KCA* (55%) and *AKT1* (13%) mutations^[70]^.

The high prevalence of PI3K pathway alterations in TNBC led to major investment in preclinical and clinical drug development targeting multiple components of the pathway, particularly AKT. Two phase 2, randomized, placebo-controlled clinical trials provided pilot clinical evidence that TNBC patients might benefit from AKT inhibition. The PAKT trial (NCT03997123)^[67]^ and LOTUS trial (NCT02162719)^[71]^ identified approximately 25% and 41% of patients with *PIK3CA*/*AKT1*/*PTEN* alterations, respectively. The differences in pathway alteration rate between the 2 trials were possibly due to different NGS assays, variant calling and ethnicity disparity. The LOTUS trial showed that adding ipatasertib, a highly selective small-molecule AKT inhibitor, to paclitaxel as first-line treatment in metastatic TNBC patients could significantly improve progression-free survival (PFS) compared with that for placebo plus paclitaxel (intent-to-treat, ITT population, PFS 6.2 months *vs.* 4.9 months, HR = 0.60, *P* = 0.037), more so in predefined *PIK3CA*/*AKT1*/ *PTEN*-altered patients characterized by next-generation sequencing (PFS 9.0 months *vs.* 4.9 months, non-stratified HR = 0.44, *P* = 0.041), but not in PTEN-low patients defined by immunohistochemistry (IHC) staining^[71]^. The most common grade ≥ 3 adverse events were diarrhea (23% of ipatasertib group *vs.* 0% of placebo group), neutrophil count decreased (8% *vs.* 6%), and neutropenia (10% *vs.* 2%) - the latter two preferred terms are synonymous, but were recorded separately in the safety database. Surprisingly, the confirmatory phase 3 IPATunity130 trial (NCT03337724) failed to confirm significant PFS improvement with the addition of ipatasertib to first-line paclitaxel in a larger cohort of 255 patients with *PIK3CA*/*AKT1*/*PTEN*-altered locally advanced unresectable or metastatic TNBC (median PFS 7.4 months *vs.* 6.1 months, HR = 1.02, 95%CI: 0.71–1.45)^[72]^. Further biomarker analyses are to be conducted to evaluate why the pivotal phase 3 trial failed to recapitulate the randomized phase 2 data. Meanwhile, the results from the randomized phase 2 PAKT trial were consistent with the LOTUS study^[67]^. Untreated metastatic TNBC patients were randomized to receive paclitaxel plus the highly selective pan-AKT inhibitor capivasertib or paclitaxel plus placebo. Statistically significant and clinically meaningful prolonged PFS from adding capivasertib was only observed in *PIK3CA*/*AKT1*/*PTEN*-altered patients (9.3 months *vs.* 3.7 months, HR = 0.30, *P* = 0.01), but neither in the ITT population nor in the *PIK3CA*/*AKT1*/*PTEN*-non-altered subgroup. Patients who received capivasertib plus paclitaxel had significantly longer OS than those with placebo plus paclitaxel in the ITT population (19.1 months *vs.* 12.6 months, HR = 0.6, *P* = 0.04). The most common severe adverse events in capivasertib group *vs.* placebo group were diarrhea (13% *vs.* 1%), infection (4% *vs.* 1%), neutropenia (3% *vs.* 3%), rash (4% *vs.* 0%), and fatigue (4% *vs.* 0%), respectively. Taken together, prospectively planned biomarker analyses support the selection of patients with *PIK3CA*/*AKT1*/*PTEN* alterations for future studies. Capivasertib is under further investigation for metastatic TNBC patients in a phase 3 randomized trial (NCT03997123).

### Targeting cell surface targets in triple-negative breast cancer by antibody-drug conjugates

Antibody-drug conjugates (ADCs) are a novel class of complex cancer therapeutics that consist of an antibody, a linker and a cytotoxic payload. The antibody backbone typically targets a cell surface protein expressed by tumor cells (and/or in the tumor microenvironment, TME). Over the past decade, with advanced technological development, ADCs have achieved substantial progress in antibody design, linker chemistries, and payload selection. At the time of this writing, three antibody-drug conjugates have been approved by the United States Food and Drug administration (FDA) for breast cancer: ado-trastuzumab emtansine (T-DM1) for metastatic and early stage HER2-amplified or overexpressing breast cancer (2013, 2019)^[73,74]^, fam-trastuzumab deruxtecan-nxki (DS-8201) for metastatic HER2-positive breast cancer (2019)^[75]^ and sacituzumab govitecan (SG, IMMU-132) for metastatic TNBC following ≥ 2 prior lines of therapy in the metastatic setting (2020)^[76]^. SG targets Trop-2, a transmembrane calcium signal transducer detected in all breast cancer subtypes, particularly in TNBC^[77]^. The cytotoxic payload, SN-38 (a potent topoisomerase I inhibitor) is coupled to the humanized anti-Trop-2 monoclonal antibody hRS7 IgG1κ through a cleavable CL2A linker, allowing for the delivery of therapeutic concentrations of the drug in both targeted cells and bystander cells. The FDA-registrational phase 1/2 single-arm IMMU-132–01 clinical trial (NCT01631552) enrolled 108 TNBC patients who have received at least two prior lines of therapy^[76]^. Single-agent 10 mg/kg SG intravenously on days 1 and 8 of each 21-day cycle was administrated to all participants until disease progression or unacceptable toxicity with an objective response rate (ORR) of 33.3% (95%CI: 24.6–43.1) and median duration of response of 7.7 months (95%CI: 4.9–10.8). The most frequent serious adverse reactions of SG (reported in > 1%) include febrile neutropenia (6%), vomiting (5%), nausea (3%), dyspnea (3%), diarrhea (4%), anemia (2%), pleural effusion (2%), neutropenia (2%), pneumonia (2%) and dehydration (2%)^[77]^. The durable objective response in heavily pretreated metastatic TNBC patients prompted the FDA’s accelerated approval of SG in early 2020. More recently, as presented at 2020 European Society of Medical Oncology meeting, results of the phase 3 confirmatory ASCENT study (NCT02574455) met its primary endpoint of PFS with statistical confidence and confirmed the initial observations in the phase 1/2 study^[78]^. In this open-label trial, 468 patients with brain metastasis-negative TNBC who previously received at least 2 prior therapies for metastatic disease were randomized 1:1 to receive SG or single-agent chemotherapy treatment per physician’s choice (TPC, capecitabine, eribulin, vinorelbine or gemcitabine). When compared with the TPC control arm, patients who received SG achieved significantly increased median PFS (5.6 months *vs.* 1.7 months, HR = 0.41, *P* < 0.0001), OS (12.1 months *vs.* 6.7 months, HR = 0.48, *P* < 0.0001) and ORR (35% *vs.* 5%, *P* < 0.0001), respectively. Severe adverse events with SG (*vs.* TPC) were neutropenia (51% *vs.* 33%), diarrhea (10.5% *vs.* < 1%), anemia (8% *vs.* 5%), and febrile neutropenia (6% *vs.* 2%). No grade > 3 neuropathy, interstitial lung disease, or treatment-related deaths were reported with SG. Notably, pre-specified exploratory analysis showed that clinical benefit with SG *vs.* TPC (PFS, OS, ORR) was irrespective of tumor Trop-2 expression level or BRCA 1/2 germline mutation status^[79]^. Other ADCs with a similar design (a widely expressed tumor surface antigen as target and antimicrotubule payload) such as ladiratuzumab vedotin targeting LIV1 (NCT04032704, NCT03310957) are still under early clinical development in TNBC. Preliminary efficacy data have shown to encourage clinical activity of ladiratuzumab vedotin in addition to pembrolizumab as first-line therapy in metastatic TNBC patients^[80]^.

Trastuzumab deruxtecan (T-DXd) is a novel HER2-targeted ADC that was designed to deliver a potent topoisomerase I inhibitor payload to HER2-expressing cancer cells with limited systemic toxicity^[81]^. On December 20, 2019, the FDA granted accelerated approval to T-DXd to metastatic HER2-positive breast cancer based on its extraordinary efficacy results and manageable safety profiles from the DESTINY-Breast01 clinical trial (NCT03248492)^[75]^. In preclinical studies, the potent antitumor activity of T-DXd was observed in low HER2-expressing breast cancer cells, probably due to bystander effects and high drug-to-antibody ratio of T-DXd^[81,82]^. Thus, T-DXd was then explored in early phase clinical trials in metastatic breast cancers defined as “HER2-low” (i.e., IHC 1–2+ and HER2 non-amplified, some of which were TNBC). A dose-escalation and expansion phase 1 study (NCT02564900) evaluated the safety and activity of T-DXd in patients with advanced HER2 low-expressing solid tumors including 47 hormone receptor-positive breast cancer patients and 7 TNBC patients^[83]^. After receiving T-DXd 5.4 or 6.4 mg/kg intravenously once every 3 weeks, patients with low HER2 expression achieved ORR of 20/54 (37.0%, 95%CI: 24.3%−51.3%) in the overall population and 1/7 (14.3%, 95%CI: 0.4%−57.9%) in the TNBC subgroup. A phase 3, randomized, multicenter study (DESTINY-Breast04, NCT03734029) has been initiated to confirm this observation, and will include a fraction of TNBC patients whose tumors are HER2-low (although the protocol-defined primary endpoint population is in hormone receptor positive patients). Likewise, SYD985, another trastuzumab-based ADC consisting of trastuzumab and a duocarmazine payload was also reported to achieve ORR of 40% and median PFS of 4.9 months in 17 HER2-low expressing TNBC patients (NCT02277717) in a phase 1 clinical trial^[84]^. If the ongoing and/or future phase 3 studies confirm the early-phase trial results reported here, HER2-targeting ADCs may provide a future novel treatment option for advanced “HER2-low” breast cancer (including TNBC) patients.

In addition to looking for suitable targets and improving the linkers’ chemistry, the evolution of ADCs also broadens the spectrum of payloads beyond antimicrotubule agents. Small payload such as molecularly targeted agents and immunostimulant agents with normal tissue toxicity or shown to be unsafe when administrated systemically as single agents, can be efficiently delivered to antigens (either on tumor cells or on stromal cells in the TME) with limited toxicities^[85]^. A select number of ADCs with promising payloads of either targeted therapies or immune stimulants are entering clinical trials^[85]^.

### Targeting androgen receptor in triple-negative breast cancer

AR-expressing TNBC cell lines and *in vivo* models have demonstrated that AR stimulation enables tumor growth while AR antagonists could inhibit tumor growth^[86–89]^. Hence, TNBC patients with abundant AR expression may benefit from pharmacologic inhibition of androgen signaling pathway. To date, very modest antitumor activity has been observed in three phase 2 clinical trials of AR inhibitors - enzalutamide, bicalutamide and abiraterone acetate (NCT00468715, NCT01842321, NCT01889238)^[90–92]^. However, due to the lack of a control arm, the clinical benefit observed in these trials could merely be derived from the better prognosis of AR-expressing TNBC patients. To address this concern, at least one randomized, phase 3 clinical trial is currently underway (NCT03055312) comparing bicalutamide *vs.* chemotherapy of physicians’ choice in first-line metastatic TNBC.

## LESSONS LEARNED FROM CURRENT IMMUNOTHERAPY STUDIES OF TRIPLE-NEGATIVE BREAST CANCER

Tumor neoantigens are accumulated during tumor evolution and continued mutational processes, which are recognized by T cells, leading to activation of antitumor immune response^[93]^. However, by the time tumors are clinically detectable, they have developed mechanisms to escape the immune surveillance through the attenuation of immune detection (for example, cGAS-STING pathway signaling^[94]^), and of T cell responses (for example, via immune checkpoint perturbation^[95]^). Cancer immunotherapy impedes the tumor’s evasion mechanisms, breaks the tolerance acquired by tumors and vitalizes the immune system to attack cancer.

### Immunogenicity and the tumor immune microenvironment

The importance of immune surveillance in determining the prognosis of various tumor types is increasingly recognized. More than 70% of TNBCs contain at least 1% tumor-infiltrating lymphocytes (TILs)^[96]^. The immune microenvironment strongly influences clinical outcomes in TNBC; indeed, the presence of TILs correlates with better prognosis in patients with early stage TNBC^[96]^. Despite more prevalent enrichment of TILs, meaningful clinical responses of immunotherapy harnessing CTLA-4 and PD(L)-1 have only been observed in a subset of TNBC patients, in contrast to practice-changing findings in melanoma, lung cancer and kidney cancer^[97]^. Two possible explanations include low immunogenicity of a sizable fraction of TNBCs, and the lack of understanding of the complexities of the immune repertoire as well as their spatial/geographic distribution and dynamic temporal trafficking of infiltrating immune cells.

By extracting immune metagene expression values, histologic lymphocyte counts and prognosis data from The Cancer Genome Atlas (TCGA) breast cancer database, Karn *et al*.^[98]^ identified an immune-rich (*n* = 25) cohort and an immune-poor (*n* = 168) in primary TNBC patients. Clonal heterogeneity, somatic total mutational load, neoantigen load, and somatic copy number alteration were compared between cohorts. High immune infiltration was frequently observed in primary TNBC with lower clonal heterogeneity, fewer somatic CNAs, and lower somatic mutation and neoantigen loads, challenging the hypothesis that cancers with greater genomic instability (such as TNBC) generate more neoantigens, leading to more significant immune infiltration. The authors inferred that high immune infiltration may reflect effective immune surveillance that continuously eliminates immunogenic clones, resulting in less clonal heterogeneity, “simpler” genomes in the surviving cancers, and lower “immunogenicity” than the corresponding primary tumor (even though causal-effect relationships cannot be established based on this observation)^[98]^.

Moreover, elucidating the complexity of immune cell phenotypes in the TME is essential for understanding the mechanisms of cancer progression and immunotherapy response. In breast cancer, significant heterogeneity in the immune composition is observed across tumor subtypes and patients^[99]^. Recent single-cell RNA sequencing and mass cytometry studies provided a glimpse into immune cell phenotypic diversity in the breast cancer microenvironment, or “ecosystem”, serving as an immune atlas in breast carcinomas^[100–102]^. Azizi *et al*.^[100]^ profiled 45,000 immune cells with single-cell RNA-seq from 8 breast carcinomas samples including TNBC, as well as matched normal breast tissue, blood, and lymph nodes and revealed continuous activation and differentiation states of T cells specific to the TME, challenging the conventional theory that TME is shaped only by few discrete states of T cell differentiation or activation. Along with a large-scale mass cytometry study from Wagner *et al*.^[102]^, both studies revealed lymphoid and myeloid cell lineages, with significantly increased intra-tumoral heterogeneity, were continuously shaped by the tumor cells and immune cells in the surrounding microenvironment, which was typically not associated with cancer immunogenicity. To better bridge the gap between more easily-accessed animal models and human clinical data, Kim *et al*.^[103]^ characterized the tumor immune infiltrate composition in TNBC mouse models and validated the results in a human breast cancer dataset. Using flow cytometry to profile the quantity of immune cells in tumors and the neutrophil-to-macrophage ratio, Kim *et al*.^[103]^ were able to identify 3 main immune subtypes: (1) a macrophage-enriched subtype (MES), in which abundant macrophage and few neutrophils infiltrate in the tumors, but lack systemic immune response; (2) a neutrophil-enriched subtype (NES), with rich neutrophil infiltration in the tumor and increased systemic immunity; and (3) an immunological cold subtype, with scant immune cell infiltration. The authors demonstrated that MES tumors responded to immunotherapeutic approaches (with varied sensitivity) whereas cold and NES tumors were immunotherapy-resistant. Emergence of acquired resistance to immunotherapy in the MES tumors was associated with a phenotypic shift from MES to NES. However, an attempt of reversing immunotherapy resistance in NES tumors with sole neutrophil elimination failed due to a surprising increase of monocytes, the precursors of macrophages. Therefore, the authors inferred that dual blockade of both neutrophils and monocytes may be warranted in NES tumor treatment. This study suggested that TNBC is able to establish an immunosuppressive microenvironment to evade immune surveillance and that the immune microenvironment may be shaped by tumors to adapt to therapeutic attacks. Hence, the studies supporting co-evolution of the tumor and tumor immune microenvironment before and during immunotherapy provide a new perspective to better understand immunotherapy resistance in TNBC treatment.

Other than focusing on the composition and functionality of tumor immune microenvironments, some researchers observed that lactic acidosis in the TME imposed by nutrient depletion during tumor progression could alter the function of anti-tumor immune cells and serve as a major driver for immune evasion in TNBC^[104]^. Two concepts emerged to potentially reverse the negative impacts of TME on anti-tumor immune cells. First, targeting the metabolism of TME may have the potential to improve cancer treatment. For example, pre-clinical evidence suggested that buffering intra-tumoral pH by oral bicarbonate therapy could inhibit tumor growth with increased CD8+ T cell infiltration in murine melanoma and pancreatic tumor models^[105]^ and could improve NK cell infiltration and IFN-γ production in a murine lymphoma model^[106]^. Efforts have also been made to generate metabolic preconditioning immune cells by adoptive T cell treatment to enhance their persistence and effector function within the glycolytic TME^[107]^.

Another intriguing observation about checkpoint inhibition treatment in TNBC is that it appears to be less effective in heavily pretreated TNBC than in untreated patients. Single-agent immune checkpoint inhibition elicits a much lower response rate (5%−6%) in the late-line setting compared with response rates of 19%−24% when administered as first-line treatment^[108,109]^. Emerging data suggest shifts of immune phenotype and abundance of tumor-infiltrating immune cells between primary and metastatic TNBC might bend the evolution of immune microenvironments in breast cancer during disease progression and lead to treatment resistance.

Szekely *et al*.^[110]^ compared TIL counts, programmed death-ligand 1 (PD-L1) protein expression by IHC, and immune gene expression profiles in paired primary and metastatic cancer samples. Seven hundred and thirty immune-related genes were grouped into 14 immune cell type metagenes and 22 immune functions. TIL counts and PD-L1 protein expression in either tumor or stromal cells were substantially lower in metastatic tumors than in primary tumors. Expression of 6 of 14 immune-cell metagene clusters and 13 of 29 potential immunotherapy targets such as PD1, PD-L1, and CTLA4 was also significantly decreased in metastatic breast cancer samples, suggesting an immune-cell-depleted and immunosuppressive microenvironment in metastasis via downregulation of chemotactic and immune-activating cytokines, and decreased antigen presentation. More recently, Hutchinson *et al*.^[111]^ applied targeted exome sequencing and whole-transcriptome sequencing to paired primary and metastatic TNBC samples, and integrated mutational information with gene expression. Overall, 50% or more of mutations were shared between primary and metastatic TNBC pairs. No significant changes in copy-number aberration or tumor mutational burden were observed between primary and metastatic TNBC pairs. In contrast to few mutational shifts observed, transcriptomic and IHC analyses revealed significantly reduced immune-activating gene expression signatures and TILs in recurrent TNBCs, which is consistent with the work from Szekely *et al*.^[110]^. Both studies support early intervention with immune checkpoint inhibition in primary TNBC or first-line metastatic settings.

Currently, there are no approved immune checkpoint inhibitors for early-stage TNBC. To address this shortcoming, the I-SPY2 study (NCT01042379) is a phase 2 platform trial designed to rapidly screen therapeutics that are likely to succeed in phase 3 trials for high-risk early breast cancer patients. In an anti-PD-1 antibody (pembrolizumab) study, 181 early TNBC patients were randomized to receive conventional NAC (paclitaxel followed by anthracycline and cyclophosphamide) and 69 to receive 4 cycles of neoadjuvant pembrolizumab in addition to NAC^[112]^. Final estimated pCR rates were 60% in the pembrolizumab arm and 22% in the control arm, indicating a high probability of success in a phase 3 confirmatory trial. Indeed, preliminary results from the phase 3 KEYNOTE 522 study (NCT03036488) and IMpassion 031 trial (NCT03197935) provide further support for this approach. In the KEYNOTE 522 trial, patients with previously untreated stage II-III TNBC were randomized 2:1 to receive NAC with 4 cycles of pembrolizumab, or placebo plus paclitaxel and carboplatin followed by 4 cycles of pembrolizumab or placebo, plus anthracycline/cyclophosphamide^[112]^. After definitive surgery, the patients continued to receive adjuvant pembrolizumab (or placebo) every 3 weeks for up to 9 cycles. Two co-primary endpoints included the pCR rate at the time of definitive surgery, and event-free survival (EFS) in the intention-to-treat population. At the first interim analysis, adding pembrolizumab to standard NAC significantly increased pCR rate from 51.2% in the placebo group to 64.8% in the pembrolizumab group (95%CI: 5.4–21.8, *P* < 0.001), irrespective of PD-L1 expression^[113]^. However, according to the recently-released results of the third planned interim analysis, the pCR difference between treatment arms has decreased to just 7.5% (63.0% *vs.* 55.6%, estimated difference 7.5%, 95%CI: 1.6–13.4), and was considered to be the most accurate estimate since it involved all patients who were randomized in KEYNOTE 522^[114]^. At the time of writing, the EFS endpoint had not met its pre-specified threshold for statistical significance and remained immature with 53% of targeted EFS events that had occurred. Considering the questionable clinical meaningfulness of the pCR rate improvement after adding pembrolizumab to conventional neoadjuvant chemotherapy, and immaturity of the EFS data, on February 9, 2021, the FDA’s Oncology Drugs Advisory Committee deferred the approval of pembrolizumab in combination with chemotherapy as neoadjuvant therapy for high-risk, early-stage TNBC, citing need for longer follow-up to capture time-to-event data^[114]^.

In the IMpassion 031 trial, previously untreated stage II-III TNBC patients were randomly assigned (1:1) to receive atezolizumab (a monoclonal antibody targeting PD-L1) or placebo with NAC comprised of nab-paclitaxel followed by anthracycline/cyclophosphamide^[115]^. Patients in the atezolizumab group were unmasked post-operatively and continued to receive 11 more cycles of atezolizumab in the adjuvant setting. Atezolizumab in combination with NAC yielded a significantly improved pCR rate of 58% when compared to the placebo group of 41% (95%CI: 6–27, *P* = 0.0044), irrespective of PD-L1 status. A tolerable safety profile from all 3 trials noted above is consistent with the known risks of the individual component study drugs^[115]^. A recent update of patient-reported outcome of the IMpassion031 trial showed that adding atezolizumab to chemotherapy improved pCR without adding treatment burden or compromising quality of life metrics for study patients^[116]^. Of note, the NeoTRIP (NCT002620280) trial, a phase 3 study that included 208 early-stage TNBC patients and compared adding atezolizumab to neoadjuvant nab-paclitaxel and carboplatin regimen, did not show a significant benefit in pCR [pCR 43.5% *vs.* 40.8%, odds ratio (OR) = 1.11, 95 %CI: 0.69–1.79, *P* = 0.66]^[117]^. Thus, it has been speculated that anthracyclines may be important to obtain the greatest benefit from immunotherapy as measured by pCR^[117,118]^.

In the GeparNuevo study (NCT02685059)^[119]^, a high proportion of patients with early-stage disease was enrolled (45% cT1 and 68% cN0) and pCR rate with durvalumab (an anti-PD-L1 antibody) was not significantly improved compared to the placebo arm of neoadjuvant nab-paclitaxel followed by anthracycline/cyclophosphamide (pCR 53.4% *vs.* 44.2%, OR = 1.45, 95%CI: 0.80–2.63). This raised the possibility that the GeparNuevo trial may be underpowered for TNBC patients with high-stage and node-positive disease, who might benefit most from immunotherapy. Despite negative findings in the general treatment population, the investigators reported an additional potentially important angle to look at the timing of immunotherapy. They found that a “run-in” period of durvalumab prior to NAC improved the pCR rate compared to concomitant therapy (pCR 61.0% *vs.* 41.4%, OR = 2.22, 95%CI: 1.06–4.64, *P* = 0.035, interaction *P* = 0.048). Further studies are warranted to reveal whether there are true immunological interactions caused by upfront single-agent immunotherapy and whether this treatment schedule could lead to improved clinical outcomes.

### Does the chemotherapy backbone matter for checkpoint inhibition in triple-negative breast cancer?

It has been shown in the early clinical trials of immune checkpoint inhibition that the majority of TNBC patients do not benefit from single-agent immune checkpoint inhibitors with ORR 5%−20% and median PFS of only 2 months^[108,109,120,121]^, thus highlighting the need for combination with other chemotherapy drugs or targeted therapies to increase the efficacy of PD(L)-1 blockade. The selection of chemotherapy backbone for novel combinatorial immunotherapy regimens is one potentially important controllable clinical variable. Currently, a growing body of evidence shows that the efficacy of conventional chemotherapy agents is derived from both direct cytotoxic activity and the (re)activation of tumor-targeting immune responses^[122]^. To investigate the theory that conventional radiation or chemotherapy may enhance the potency of checkpoint inhibitor via inducing T cell priming, the phase 2 TONIC (NCT02499367) study was conducted to characterize immunomodulatory effects of hypo-fractionated irradiation, low-dose cyclophosphamide, cisplatin and doxorubicin in metastatic TNBC patients^[118]^. Sixty-seven patients were randomized to receive 1 of 4 induction treatments for 2 weeks (the treatment cohort) or a 2-week waiting period (the waiting cohort) followed by nivolumab, a PD-1 inhibitor. The majority of responses were observed in the doxorubicin (ORR 35%) and cisplatin (ORR 23%) cohorts. After doxorubicin and cisplatin induction, an upregulation of immune-related genes involved in PD(L)-1 and T cell cytotoxicity pathways were also detected. A trend towards increased T cell infiltration and TCR diversity appeared more strongly in the doxorubicin cohorts after nivolumab treatment than in the waiting cohort. Based on a Simon two-stage statistical design, the doxorubicin cohort is currently under expansion in stage II of the trial. Another example comes from the IMpassion 130 trial (NCT02425891) and the IMpassion 131 trial (NCT03125902). Atezolizumab, combined with nab-paclitaxel, is the first immunotherapy for breast cancer that received accelerated approval from the United States FDA in March 2019, for patients with advanced PD-L1-expressing TNBC. A statistically significant improvement of median PFS (7.5 months with atezolizumab and nab-paclitaxel and 5.0 months with placebo and nab-paclitaxel, HR = 0.62, 95%CI: 0.49–0.78, *P* < 0.001) was observed in the patients with PD-L1 expression on tumor-infiltrating immune cells (PD-L1 positive subgroup)^[123]^. More recently, a final median overall survival improvement of 7.5 months was also reported in the PD-L1 positive subgroup (25.4 months *vs.* 17.9 months, HR = 0.67, 95%CI: 0.53–0.86)^[124]^. However, in the “confirmatory” phase 3 trial (IMpassion 131) with identical patient inclusion criteria and trial design but different chemotherapy formulation as the backbone (i.e., cremophor-formulated paclitaxel as opposed to albumin-formulated nab-paclitaxel), surprisingly, neither PFS nor OS improvement was observed in the intention-to-treat population or in the PD-L1 positive subgroup when atezolizumab plus paclitaxel was compared to placebo plus paclitaxel^[125]^. We can speculate that cremophor-based paclitaxel may not induce tumor-targeting immune responses as much as nab-paclitaxel does as a result of the requirement for steroid premedication. However, this notion is not supported by the KEYNOTE 355 pembrolizumab phase 3 study (NCT02819518), in which subgroup analysis indicated that both cremophor-paclitaxel and nab-paclitaxel appeared similarly efficacious when combined with pembrolizumab^[126]^. In November 2020, the FDA granted accelerated approval to pembrolizumab in combination with chemotherapy to treat patients with locally recurrent unresectable or metastatic TNBC, whose tumors express PD-L1 [using a different PD-L1 detection antibody and different scoring criteria - combined positive score (CPS) ≥ 10, as compared to the PD-L1 reagents used for the atezolizumab TNBC trials]. The approval was based on KEYNOTE-355 trial^[127]^, a phase 3 randomized, placebo-controlled clinical trial conducted in previously untreated locally recurrent or metastatic TNBC patients. Patients were randomized 2:1 to pembrolizumab plus chemotherapy *vs.* placebo plus chemotherapy. The chemotherapy backbone in this trial included nab-paclitaxel, paclitaxel, or gemcitabine plus carboplatin. In the PD-L1 positive subgroup (CPS ≥ 10, cf. below), adding pembrolizumab to chemotherapy of choice extended median PFS from 5.6 to 9.7 months (HR = 0.65, 95%CI: 0.49–0.86, *P* = 0.0012), meeting one of the protocol-defined primary endpoints. Results for the key secondary endpoints of ORR, duration of response and disease control rate all favored the pembrolizumab-randomized group, with the treatment effect increasing as CPS increased^[127]^. However, the trial was not adequately powered to critically compare efficacy among treatment groups by different chemotherapy regimens.

### Predictive biomarkers for response to PD-(L)1 checkpoint inhibition in triple-negative breast cancer

Until now, PD-L1 is the only FDA-approved predictive biomarker for immune checkpoint inhibitors treatment in metastatic TNBC. As pointed out above, different commercial diagnostic assays, scoring systems and definitions of PD-L1 positivity were applied in various immune checkpoint inhibitor clinical trials. Two commercially available diagnostic assays, the VENTANA PD-L1 (SP142) assay and the PD-L1 IHC 22C3 pharmDx assay have been approved as companion diagnostics for selecting TNBC patients to receive atezolizumab and pembrolizumab, respectively. In the neoadjuvant KEYNOTE 522 trial, the benefit of pembrolizumab-chemotherapy in terms of pCR was generally consistent across subgroups, including PD-L1 positive subgroups. In this trial, PD-L1 expression was assessed by the PD-L1 IHC 22C3 pharmDx assay and was determined with CPS, defined as the number of PD-L1-positive tumor cells, lymphocytes, and macrophages divided by the total number of tumor cells multiplied by 100; specimens with a CPS ≥ 1 were considered PD-L1-positive^[113]^. In contrast, in the metastatic KEYNOTE 355 trial, using the same diagnostic assay and scoring system, the boundary for a statistically significant benefit of pembrolizumab-chemotherapy in patients with CPS ≥ 1 tumor was not met (but was met when CPS ≥ 10)^[127]^. These findings differed from the results of the IMpassion 130 trial, which showed the benefit of atezolizumab only held in patients with PD-L1 positive metastatic TNBC defined as the expression on tumor-infiltrating immune cells ≥ 1% by SP142 assay. Collectively, the predictive value of PD-L1 expression for checkpoint inhibitors remains controversial given the variable IHC antibodies, absence of a unified scoring system, and discordance for scoring PD-L1 expression on different cell compartments (summarized in Table 2). Moreover, discordance of PD-L1 expression between results from SP142 assay and 22C3 assay has been reported via head-to-head comparisons in TNBC patient specimens^[128,129]^, despite earlier published data suggesting these two assays were analytically concordant, indicating that they may even be interchangeable^[130]^. Finally, since PD-1/PD-L1 interaction is only one of many pathways exploited by anti-tumor immunity, it seems unlikely that one sole biomarker could sufficiently predict clinical response to immune checkpoint inhibitors in different treatment settings^[131]^, particularly in the neoadjuvant setting where efficacy signals from checkpoint inhibition in TNBC appear to be irrespective of PD-(L)1 immunostaining status.

Also, some studies with limited sample sizes have suggested an association between high TMB and improved clinical benefit of CTLA-4 and PD-(L)1 inhibitors in multiple cancers. Samstein *et al*.^[132]^ analyzed the clinical and genomic data of 1662 advanced cancer patients treated with immune checkpoint inhibitors and 5371 non-immune checkpoint inhibitors-treated patients. TMB was identified via targeted next-generation sequencing. Among this large cohort, 45 breast cancer patients were included, yet TMB did not predict OS in breast cancer patients treated with immune checkpoint inhibitors. More recently, Barroso-Sousa *et al*.^[133]^ applied targeted WES to 62 metastatic TNBC samples from patients who were treated with anti-PD(L)-1 therapies. In this study, high TMB was associated with improved PFS (12.5 months *vs.* 3.7 months, *P* = 0.04) but not OS. Future studies need to be conducted in larger datasets. Given small sample sizes and inconsistent results of predictive markers studies across different cancer types, Lu *et al*.^[134]^ performed a meta-analysis to examine a range of biomarkers such as IHC expression of PD-L1 (PD-L1 IHC), TMB, GEP, and multiplex immunohistochemistry/immunofluorescence (mIHC/IF) assays, alone or combined, and determine their relationships with objective response to anti-PD-(L)1therapies. In all, 8135 patients covering 10 tumor types were analyzed in this meta-analysis. TMB, PD-L1 IHC, and GEP showed comparable sensitivity and specificity in predicting response to immunotherapy, whereas mIHC/IF and multimodality biomarker strategies demonstrated better predictive values; however, these findings need future prospective validation.

**Figure 1.**
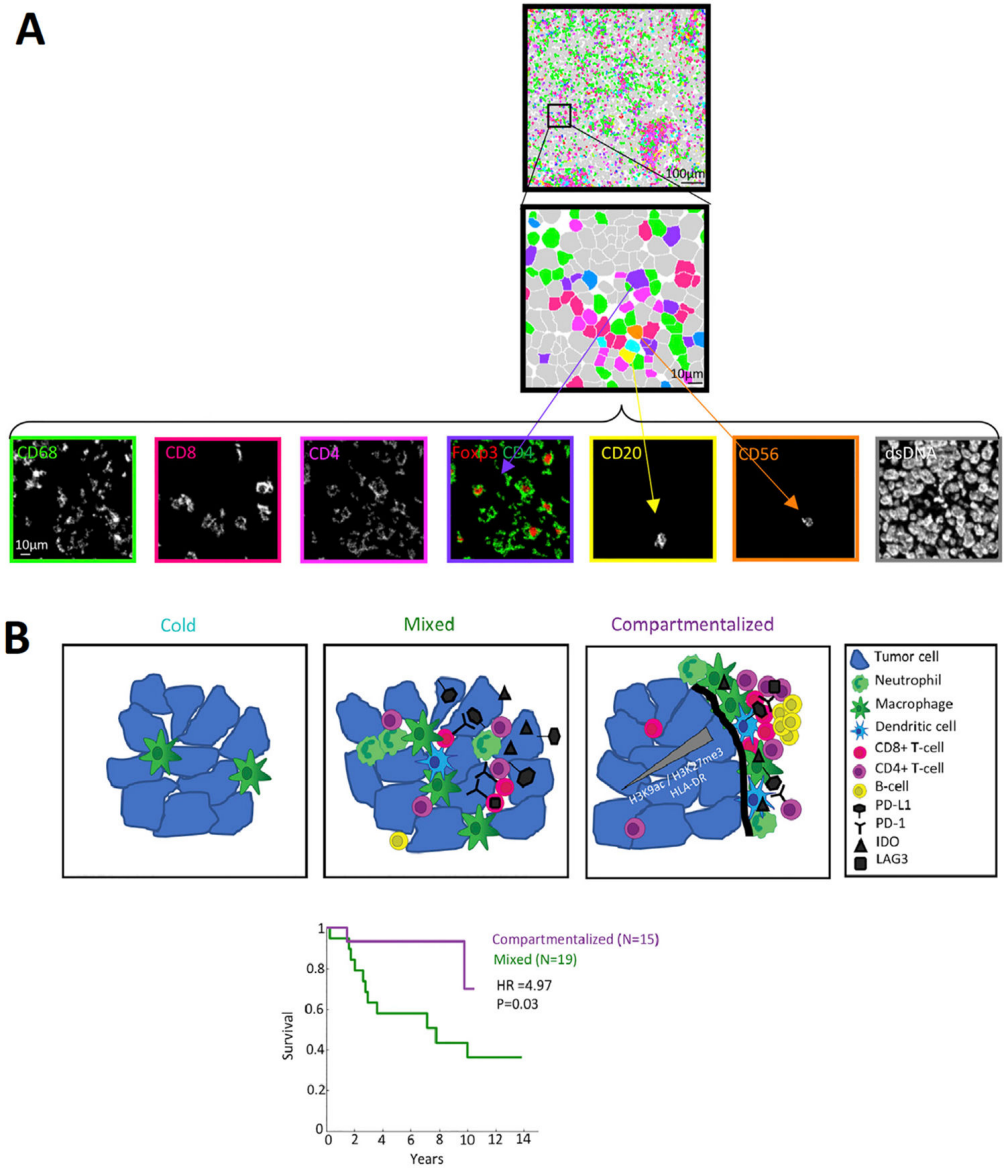
Automated image analysis pipeline delineates ordered immune composition in TNBC, using MIBI-TOF. A: Top: Pseudo-coloring of tumor-infiltrating immune cells in a patient with TNBC. Bottom: Expression of 7 markers demonstrating the repertoire of infiltrating immune cells as well as their spatial location, including cell-cell contacts; B: Top: Cartoon depicting 3 archetypes of tumor-immune composition and organization in TNBC. Cold tumors have few immune cells, mainly macrophages. Mixed tumors have an admixture of tumor and immune cells. IDO and PD-L1, if expressed, are expressed primarily on tumor cells and PD-1 on CD8+ T cells. In compartmentalized tumors, the immune and tumor cells are spatially segregated. Neutrophils are enriched near the border, whereas B cells form secondary lymphoid structures further away. IDO and PD-L1 are expressed primarily on immune cells and PD-1 on CD4+ T cells. Bottom: Kaplan-Meier analysis showing survival as a function of time for patients with compartmentalized or mixed tumor-immune organizations. This figure is adapted with permission from Keren *et al*.^[135]^.Copyright 2018 by Elsevier.

Given the lack of definitive biomarkers to predict clinical benefit from immune checkpoint inhibition, Keren *et al*.^[135]^ used multiplexed ion beam imaging by time-of-flight (MIBI-TOF) to simultaneously quantify *in situ* expression of 36 proteins covering identity, function, and immune regulation at sub-cellular resolution in 41 TNBC patients [Figure 1A]. In this investigation, spatial enrichment analysis showed immune mixed and compartmentalized tumors, coinciding with expression of PD1, PD-L1, and IDO in a cell-type- and location-specific manner. Remarkably, in this small pilot study, spatial organization of immune phenotypes within triple-negative breast tumors was linked to survival [Figure 1B]. The data demonstrate organization in the tumor-immune microenvironment that is structured in cellular composition, spatial arrangement, and regulatory-protein expression, providing a framework to apply multiplexed imaging to immune oncology. CO-Detection by indexing (CODEX) technology, where each detection antibody is labeled with a unique oligonucleotide barcode, is another platform which allows simultaneous visualization of up to 40 (or more) antigens in a single tissue section, resolves the relative expression and abundance at the spatial level, thereby enabling a systems-level approach to the analysis of tissue architecture, including tumor-infiltrating immune repertoires^[136]^. Application of such technologies to characterize complete intra-tumoral immune repertoires, including how they may change dynamically before and following immunotherapeutic manipulation, will further enlighten understanding of immune biomarkers predictive for anti-tumor immune response in TNBC.

Serial analysis of liquid biopsies harnessing circulating tumor DNA (ctDNA) measurements in peripheral blood plasma allows us to look into cancer-specific somatic mutations without undergoing repetitive tissue biopsies. It has been reported that serial personalized ctDNA analysis targeting 16 variants selected from whole-exome data of individual primary tumors could predict clinical cancer relapse in primary breast cancer patients with the sensitivity of 89% and specificity of 100%, and a median lead time of 8.9 months^[137]^. Bratman *et al*.^[138]^ then conducted the INSPIRE trial (NCT02644369), a phase 2 study evaluating response of pembrolizumab in patients with advanced solid tumors (including TNBC), and using personalized ctDNA analysis to determine tumor burden. In this study, baseline ctDNA concentration was correlated with PFS, OS, ORR and clinical benefit in TNBC. An early reduction in ctDNA after 2 cycles of pembrolizumab treatment and on-treatment ctDNA clearance were effective predictive factors for good prognosis, irrespective of tumor type, TMB or PD-L1 status. This study provides a new perspective on cancer surveillance and treatment response evaluation in cancer patients treated with immunotherapy. Finally, noncoding RNAs, including microRNA (miRNA) and long-noncoding RNA (lncRNA), are considered as novel sources of prognostic and predictive biomarkers in TNBC and have been evaluated in both tissue specimens and as circulating miRNAs^[139]^. Certain circulating miRNAs were reported to be interacting with checkpoint genes involved in the immune response, which could be assessed in TNBC patients treated with immunotherapy as response predictive factors^[140]^. Moreover, subtypes based on immune-functional lncRNA signatures were shown to have strong prognostic value in bladder cancer and melanoma patients receiving immunotherapy^[141]^. Future studies could evaluate the use of immune-functional lncRNA as predictive biomarkers for PD-(L)1 checkpoint inhibition in TNBC patients.

## CONCLUSION

TNBC is merely an operational term (hopefully soon to be replaced) to define cohorts of human breast cancers based upon what they are not, rather than what they actually are - which is clearly a collection of multiple distinct disease entities based upon genomic and transcriptomic characterization, each with prognostic as well as predictive implications for response to a variety of therapeutic drug classes, ranging from DNA damaging agents, to PARP inhibition, to immunotherapeutic agents, to ADCs, and potentially to hormonal manipulation of the androgen receptor. Advances in genomics and molecular profiling have helped better define subtypes of TNBC with distinct phenotypes and biologic drivers, which still largely awaits implementation into the current clinical therapeutic armamentarium. The advent of next-generation sequencing has revealed the genomic evolution during TNBC disease progression, as well as underlying clonal dynamics and resistance mechanisms. In theory, early identification of pre-existing aggressive treatment-resistant subclones and constraining evolutionary trajectory with targeted therapeutics may impede the evolution of tumor cells with the potential for future clinical relapse and disease progression. A rich body of evidence has dissected novel biomarkers in TNBC, identified several druggable targets, and facilitated new drug development and clinical trial design. There is an ongoing exponential increase in interest in basic, translational, and clinical research in immunotherapy for TNBC. Despite the dawn of immunotherapy in TNBC, durable responses are limited to a small subset of patients, for which definitive predictive biomarkers are still lacking. Thus, the success of future clinical trials will depend on the use of new technologies such as MIBI-TOF and CODEX to decipher the heterogeneity of TNBC and its complex tumor-tumor immune microenvironment interactions.

## Figures and Tables

**Table 1.** Molecular subtypes of triple-negative breast cancer by Lehmann *et al.*^[17]^

TNBC subtype	Gene Ontologies	Differential gene expression	Therapeutic targets/drugs

Basal-like 1	Cell cycle pathway DNA damage response (ATR/BRCA pathway)	DNA damage response genes	Cisplatin PARP inhibitor
Basal-like 2	Growth factor signaling pathway Glycolysis/ Gluconeogenesis	Myoepithelial markers	Cisplatin PARP inhibitor
Immunomodulatory	Immune cell signaling pathway Cytokine signaling Antigen processing and presentation Signaling through core immune signal transduction pathways	Immune signal transduction Immune cell-surface antigens Cytokine signaling Complement cascade Chemokine receptors ligands Antigen presentation	Immune checkpoint inhibitors
Mesenchymal-like	Cell motility ECM receptor interaction Cell differentiation pathways	TGF-β, EMT-associated, growth factors signaling pathway components	PI3K/mTOR inhibitor Src inhibitor
Mesenchymal stem-like	Cell motility Cell differentiation pathways Growth factor signaling pathways	Enriched MSC-specific markers Low expression of claudins 3, 4, 7	PI3K/mTOR inhibitor Src inhibitor
Luminal androgen receptor	Hormonally regulated pathways	AR and downstream AR targets and coactivators	AR antagonist PI3K/mTOR inhibitor

AR: Androgen receptor; ATR: ATM and Rad3-related; ECM: extracellular matrix; EMT: epithelial mesenchymal transition; MSC: mesenchymal stem cell; PARP: poly ADP ribose polymerase; TGF-β: transforming growth factor beta; TNBC: triple-negative breast cancer.

**Table 2.** Summary of Phase 3 clinical trials of immune checkpoint inhibition for triple negative breast cancer in the neoadjuvant and metastatic settings

	Early-stage TNBC Neoadjuvant clinical trials	Metastatic TNBC Metastatic clinical trials

Assay used for PD-L1 expression detection	**KEYNOTE 522**^[113]^: 22C3 **IMpassion 031**^[115]^: SP142	**KEYNOTE 355**^[127]^: 22C3 **IMpassion 130**^[123]^, **IMpassion 131**^[125]^: SP142
Scored area	**KEYNOTE 522:** TC, lymphocytes, and macrophages **IMpassion 031:** tumor-infiltrating IC	**KEYNOTE 355:** TC, lymphocytes, and macrophages **IMpassion 130, IMpassion 131:** tumor-infiltrating IC
Definition of PD-L1 positivity	**KEYNOTE 522:** CPS = PD-L1 + cells (TC+IC)/TC*100 ≥ 1 **IMpassion 031:** PD-L1 + IC/tumor area (IC%) ≥ 1%	**KEYNOTE 355:** CPS ≥ 1, CPS ≥ 10 **IMpassion 130, IMpassion 131:** IC% ≥ 1%
Experimental immune checkpoint antibody	**KEYNOTE 522:** Pembrolizumab **IMpassion 031:** Atezolizumab	**KEYNOTE 355:** Pembrolizumab **IMpassion 130, IMpassion 131:** Atezolizumab
Chemotherapy backbone	**KEYNOTE 522:** PCb-EC/AC **IMpassion 031:** nab-paclitaxel-AC	**KEYNOTE 355:** nab-paclitaxel; paclitaxel; or GCb **IMpassion 130:** nab-paclitaxel **IMpassion 131:** paclitaxel
Premedication with corticosteroids	**KEYNOTE 522:** Yes **IMpassion 031:** No	**KEYNOTE 355:** Yes in the paclitaxel arm **IMpassion 130:** No **IMpassion 131:** Yes
Primary endpoint (Experimental group *vs.* Control group)	**KEYNOTE 522:** pCR (ypT0/Tis ypN0) ITT population: 64.8% *vs.* 51.2%, rate difference 13.6 (95%CI: 5.4–21.8), *P* < 0.001 PD-L1 + subgroup: 68.9% *vs.* 54.9%, rate difference 14.2 (95%CI: 5.3–23.1) **IMpassion 031:** pCR (ypT0/Tis ypN0) ITT population: 58% *vs.* 41% rate difference 17 (95%Ci: 6–27), one-sided *P* = 0.0044 (significance boundary 0·0184) PD-L1 + subgroup: 69% *vs.* 49%, rate difference 20 (95%CI: 4–35), interactive *P* = 0.52	**KEYNOTE 355:** PFS ITT population: 7.5 months *vs.* 5.6 months, HR = 0.82 (95%CI: 0.69–0.97), *P* value not available CPS ≥ 10 subgroup: 9.7 months *vs.* 5.6 months, HR = 0.65 (95%CI: 0.49–0.86), *P* = 0.0012 (significance boundary 0.00411) CPS ≥ 1 subgroup: 7.6 months *vs.* 5.6 months, HR = 0.74, (95%CI: 0.61–0.90), *P* = 0.0014 (significance boundary 0.00111) **IMpassion 130:** PFS ITT population: 7.2 months *vs.* 5.5 months, HR = 0.80 (95%CI: 0.69–0.92), *P* = 0.002 PD-L1 + subgroup: 7.5 months *vs.* 5.0 months, HR = 0.64 (95%CI: 0.51–0.80), *P* < 0.001 **IMpassion 131:** PFS ITT population: 5.7 months *vs.* 5.6 months, HR = 0.86 (95%CI: 0.70–1.05), *P* value not available PD-L1 + subgroup: 6.0 months *vs.* 5.7 months, HR = 0.82 (95%CI: 0.60–1.12), *P* = 0.20
Primary clinical outcome dependent upon PD-L1 expression	**KEYNOTE 522:** No **IMpassion 031:** No	**KEYNOTE 355:** CPS ≥ 1, No; CPS ≥ 10, Yes **IMpassion 130:** Yes **IMpassion 131:** No

TNBC: Triple negative breast cancer; TC: tumor cells; IC: immune cells; CPS: combined positive score; PCb: paclitaxel plus carboplatin; AC: doxorubicin plus cyclophosphamide; EC: epirubicin plus cyclophosphamide; GCb: gemcitabine plus carboplatin; pCR: pathological complete response; CI: confidence intervals; PFS: progression-free survival; ITT: intention-to-treat; HR: hazard ratio; PD-L1: programmed death-ligand 1.

## References

[1] Perou CM, Sørlie T, Eisen MB, Molecular portraits of human breast tumours. Nature 2000;406:747–52. 10963602 10.1038/35021093

[2] Wang C, Kar S, Lai X, Triple negative breast cancer in Asia: An insider’s view. Cancer Treat Rev 2018;62:29–38. 29154023 10.1016/j.ctrv.2017.10.014

[3] Thakur KK, Bordoloi D, Kunnumakkara AB. Alarming burden of triple-negative breast cancer in India. Clin Breast Cancer 2018;18:e393–9. 28801156 10.1016/j.clbc.2017.07.013

[4] Wolff AC, Hammond MEH, Allison KH, Human epidermal growth factor receptor 2 testing in breast cancer: American Society of Clinical Oncology/College of American Pathologists Clinical Practice Guideline Focused Update. Arch Pathol Lab Med 2018;142:1364–82. 29846104 10.5858/arpa.2018-0902-SA

[5] Hammond ME, Hayes DF, Dowsett M, American Society of Clinical Oncology/College of American Pathologists guideline recommendations for immunohistochemical testing of estrogen and progesterone receptors in breast cancer. J Clin Oncol 2010;28:2784–95. 20404251 10.1200/JCO.2009.25.6529 PMC2881855

[6] Foulkes WD, Smith IE, Reis-Filho JS. Triple-negative breast cancer. N Engl J Med 2010;363:1938–48. 21067385 10.1056/NEJMra1001389

[7] Liedtke C, Mazouni C, Hess KR, Response to neoadjuvant therapy and long-term survival in patients with triple-negative breast cancer. J Clin Oncol 2008;26:1275–81. 18250347 10.1200/JCO.2007.14.4147

[8] Carey LA, Dees EC, Sawyer L, The triple negative paradox: primary tumor chemosensitivity of breast cancer subtypes. Clin Cancer Res 2007;13:2329–34. 17438091 10.1158/1078-0432.CCR-06-1109

[9] Lin NU, Vanderplas A, Hughes ME, Clinicopathologic features, patterns of recurrence, and survival among women with triple-negative breast cancer in the National Comprehensive Cancer Network. Cancer 2012;118:5463–72. 22544643 10.1002/cncr.27581 PMC3611659

[10] Dent R, Trudeau M, Pritchard KI, Triple-negative breast cancer: clinical features and patterns of recurrence. Clin Cancer Res 2007;13:4429–34. 17671126 10.1158/1078-0432.CCR-06-3045

[11] Cortazar P, Zhang LJ, Untch M, Pathological complete response and long-term clinical benefit in breast cancer: the CTNeoBC pooled analysis. Lancet 2014;384:164–72. 24529560 10.1016/S0140-6736(13)62422-8

[12] von Minckwitz G, Schneeweiss A, Loibl S, Neoadjuvant carboplatin in patients with triple-negative and HER2-positive early breast cancer (GeparSixto; GBG 66): a randomised phase 2 trial. Lancet Oncol 2014;15:747–56. 24794243 10.1016/S1470-2045(14)70160-3

[13] Sikov WM, Berry DA, Perou CM, Impact of the addition of carboplatin and/or bevacizumab to neoadjuvant once-per-week paclitaxel followed by dose-dense doxorubicin and cyclophosphamide on pathologic complete response rates in stage II to III triple-negative breast cancer: CALGB 40603 (Alliance). J Clin Oncol 2015;33:13–21. 25092775 10.1200/JCO.2014.57.0572 PMC4268249

[14] Herschkowitz JI, Simin K, Weigman VJ, Identification of conserved gene expression features between murine mammary carcinoma models and human breast tumors. Genome Biol 2007;8:R76. 17493263 10.1186/gb-2007-8-5-r76 PMC1929138

[15] Prat A, Parker JS, Karginova O, Phenotypic and molecular characterization of the claudin-low intrinsic subtype of breast cancer. Breast Cancer Res 2010;12:R68. 20813035 10.1186/bcr2635 PMC3096954

[16] Prat A, Perou CM. Deconstructing the molecular portraits of breast cancer. Mol Oncol 2011;5:5–23. 21147047 10.1016/j.molonc.2010.11.003 PMC5528267

[17] Lehmann BD, Bauer JA, Chen X, Identification of human triple-negative breast cancer subtypes and preclinical models for selection of targeted therapies. J Clin Invest 2011;121:2750–67. 21633166 10.1172/JCI45014 PMC3127435

[18] Masuda H, Baggerly KA, Wang Y, Differential response to neoadjuvant chemotherapy among 7 triple-negative breast cancer molecular subtypes. Clin Cancer Res 2013;19:5533–40. 23948975 10.1158/1078-0432.CCR-13-0799 PMC3813597

[19] Lehmann BD, Jovanović B, Chen X, Refinement of Triple-negative breast cancer molecular subtypes: Implications for neoadjuvant chemotherapy selection. PLoS One 2016;11:e0157368. 27310713 10.1371/journal.pone.0157368 PMC4911051

[20] Burstein MD, Tsimelzon A, Poage GM, Comprehensive genomic analysis identifies novel subtypes and targets of triple-negative breast cancer. Clin Cancer Res 2015;21:1688–98. 25208879 10.1158/1078-0432.CCR-14-0432 PMC4362882

[21] Koboldt DC, Steinberg KM, Larson DE, The next-generation sequencing revolution and its impact on genomics. Cell 2013;155:27–38. 24074859 10.1016/j.cell.2013.09.006 PMC3969849

[22] Shah SP, Roth A, Goya R, The clonal and mutational evolution spectrum of primary triple-negative breast cancers. Nature 2012;486:395–9. 22495314 10.1038/nature10933 PMC3863681

[23] Wang Y, Waters J, Leung ML, Clonal evolution in breast cancer revealed by single nucleus genome sequencing. Nature 2014;512:155–60. 25079324 10.1038/nature13600 PMC4158312

[24] Yates LR, Gerstung M, Knappskog S, Subclonal diversification of primary breast cancer revealed by multiregion sequencing. Nat Med 2015;21:751–9. 26099045 10.1038/nm.3886 PMC4500826

[25] Gao R, Davis A, McDonald TO, Punctuated copy number evolution and clonal stasis in triple-negative breast cancer. Nat Genet 2016;48:1119–30. 27526321 10.1038/ng.3641 PMC5042845

[26] Sottoriva A, Kang H, Ma Z, A Big Bang model of human colorectal tumor growth. Nat Genet 2015;47:209–16. 25665006 10.1038/ng.3214 PMC4575589

[27] Williams MJ, Werner B, Barnes CP, Identification of neutral tumor evolution across cancer types. Nat Genet 2016;48:238–44. 26780609 10.1038/ng.3489 PMC4934603

[28] Ng C, Bidard FC, Piscuoglio S, Genetic heterogeneity in therapy-naïve synchronous primary breast cancers and their metastases. Clin Cancer Res 2017;23:4402–15. 28351929 10.1158/1078-0432.CCR-16-3115 PMC5768571

[29] Bertucci F, Ng CKY, Patsouris A, Genomic characterization of metastatic breast cancers. Nature 2019;569:560–4. 31118521 10.1038/s41586-019-1056-z

[30] Angus L, Smid M, Wilting SM, The genomic landscape of metastatic breast cancer highlights changes in mutation and signature frequencies. Nat Genet 2019;51:1450–8. 31570896 10.1038/s41588-019-0507-7 PMC6858873

[31] Nik-Zainal S, Davies H, Staaf J, Landscape of somatic mutations in 560 breast cancer whole-genome sequences. Nature 2016;534:47–54. 27135926 10.1038/nature17676 PMC4910866

[32] Balko JM, Cook RS, Vaught DB, Profiling of residual breast cancers after neoadjuvant chemotherapy identifies DUSP4 deficiency as a mechanism of drug resistance. Nat Med 2012;18:1052–9. 22683778 10.1038/nm.2795 PMC3693569

[33] Almendro V, Cheng YK, Randles A, Inference of tumor evolution during chemotherapy by computational modeling and in situ analysis of genetic and phenotypic cellular diversity. Cell Rep 2014;6:514–27. 24462293 10.1016/j.celrep.2013.12.041 PMC3928845

[34] Al-Hajj M, Wicha MS, Benito-Hernandez A, Prospective identification of tumorigenic breast cancer cells. Proc Natl Acad Sci U S A 2003;100:3983–8. 12629218 10.1073/pnas.0530291100 PMC153034

[35] Wright MH, Robles AI, Herschkowitz JI, Molecular analysis reveals heterogeneity of mouse mammary tumors conditionally mutant for Brca1. Mol Cancer 2008;7:29. 18394172 10.1186/1476-4598-7-29 PMC2329667

[36] Kim C, Gao R, Sei E, Chemoresistance evolution in triple-negative breast cancer delineated by single-cell sequencing. Cell 2018;173:879–93.e13. 29681456 10.1016/j.cell.2018.03.041 PMC6132060

[37] O’Shaughnessy J, Brezden-Masley C, Cazzaniga M, Prevalence of germline BRCA mutations in HER2-negative metastatic breast cancer: global results from the real-world, observational BREAKOUT study. Breast Cancer Res 2020;22:114. 33109210 10.1186/s13058-020-01349-9 PMC7590609

[38] Sharma P, Klemp JR, Kimler BF, Germline BRCA mutation evaluation in a prospective triple-negative breast cancer registry: implications for hereditary breast and/or ovarian cancer syndrome testing. Breast Cancer Res Treat 2014;145:707–14. 24807107 10.1007/s10549-014-2980-0 PMC4171847

[39] Ray Chaudhuri A, Callen E, Ding X, Replication fork stability confers chemoresistance in BRCA-deficient cells. Nature 2016;535:382–7. 27443740 10.1038/nature18325 PMC4959813

[40] Hahnen E, Lederer B, Hauke J, Germline mutation status, pathological complete response, and disease-free survival in triple-negative breast cancer: Secondary analysis of the GeparSixto randomized clinical trial. JAMA Oncol 2017;3:1378–85. 28715532 10.1001/jamaoncol.2017.1007 PMC5710508

[41] Tutt A, Tovey H, Cheang M, Carboplatin in BRCA1/2-mutated and triple-negative breast cancer BRCAness subgroups: the TNT Trial. Nat Med 2018;24:628–37. 29713086 10.1038/s41591-018-0009-7 PMC6372067

[42] Robson M, Im SA, Senkus E, Olaparib for metastatic breast cancer in patients with a germline BRCA mutation. N Engl J Med 2017;377:523–33. 28578601 10.1056/NEJMoa1706450

[43] IDMC has concluded that OlympiA trial of Lynparza crossed superiority boundary for invasive disease-free survival vs. placebo at planned interim analysis. Available from: https://www.astrazeneca.com/media-centre/press-releases/2021/olympia-trial-of-lynparza-idmc-recommend-early-analysis.html [Last accessed on 24 Mar 2021].

[44] Silver DP, Richardson AL, Eklund AC, Efficacy of neoadjuvant Cisplatin in triple-negative breast cancer. J Clin Oncol 2010;28:1145–53. 20100965 10.1200/JCO.2009.22.4725 PMC2834466

[45] Akashi-Tanaka S, Watanabe C, Takamaru T, BRCAness predicts resistance to taxane-containing regimens in triple negative breast cancer during neoadjuvant chemotherapy. Clin Breast Cancer 2015;15:80–5. 25445419 10.1016/j.clbc.2014.08.003

[46] Winter C, Nilsson MP, Olsson E, Targeted sequencing of BRCA1 and BRCA2 across a large unselected breast cancer cohort suggests that one-third of mutations are somatic. Ann Oncol 2016;27:1532–8. 27194814 10.1093/annonc/mdw209 PMC4959927

[47] Abkevich V, Timms KM, Hennessy BT, Patterns of genomic loss of heterozygosity predict homologous recombination repair defects in epithelial ovarian cancer. Br J Cancer 2012;107:1776–82. 23047548 10.1038/bjc.2012.451 PMC3493866

[48] Birkbak NJ, Wang Z, Kim JY, Telomeric allelic imbalance indicates defective DNA repair and sensitivity to DNA-damaging agents. Cancer Discov 2012;2:366–75. 22576213 10.1158/2159-8290.CD-11-0206 PMC3806629

[49] Popova T, Manié E, Rieunier G, Ploidy and large-scale genomic instability consistently identify basal-like breast carcinomas with BRCA1/2 inactivation. Cancer Res 2012;72:5454–62. 22933060 10.1158/0008-5472.CAN-12-1470

[50] Davies H, Glodzik D, Morganella S, HRDetect is a predictor of BRCA1 and BRCA2 deficiency based on mutational signatures. Nat Med 2017;23:517–25. 28288110 10.1038/nm.4292 PMC5833945

[51] Esteller M, Silva JM, Dominguez G, Promoter hypermethylation and BRCA1 inactivation in sporadic breast and ovarian tumors. J Natl Cancer Inst 2000;92:564–9. 10749912 10.1093/jnci/92.7.564

[52] Saal LH, Gruvberger-Saal SK, Persson C, Recurrent gross mutations of the PTEN tumor suppressor gene in breast cancers with deficient DSB repair. Nat Genet 2008;40:102–7. 18066063 10.1038/ng.2007.39 PMC3018354

[53] Turner NC, Reis-Filho JS, Russell AM, BRCA1 dysfunction in sporadic basal-like breast cancer. Oncogene 2007;26:2126–32. 17016441 10.1038/sj.onc.1210014

[54] Timms KM, Abkevich V, Hughes E, Association of BRCA1/2 defects with genomic scores predictive of DNA damage repair deficiency among breast cancer subtypes. Breast Cancer Res 2014;16:475. 25475740 10.1186/s13058-014-0475-x PMC4308910

[55] Mayer EL, Abramson V, Jankowitz R, TBCRC 030: a phase II study of preoperative cisplatin versus paclitaxel in triple-negative breast cancer: evaluating the homologous recombination deficiency (HRD) biomarker. Ann Oncol 2020;31:1518–25. 32798689 10.1016/j.annonc.2020.08.2064 PMC8437015

[56] Telli ML, Metzger O, Timms K, Evaluation of homologous recombination deficiency (HRD) status with pathological response to carboplatin +/− veliparib in BrighTNess, a randomized phase 3 study in early stage TNBC. J Clin Oncol 2018;36:519.

[57] Loibl S, Weber KE, Timms KM, Survival analysis of carboplatin added to an anthracycline/taxane-based neoadjuvant chemotherapy and HRD score as predictor of response-final results from GeparSixto. Ann Oncol 2018;29:2341–7. 30335131 10.1093/annonc/mdy460

[58] Telli ML, Hellyer J, Audeh W, Homologous recombination deficiency (HRD) status predicts response to standard neoadjuvant chemotherapy in patients with triple-negative or BRCA1/2 mutation-associated breast cancer. Breast Cancer Res Treat 2018;168:625–30. 29275435 10.1007/s10549-017-4624-7

[59] Telli ML, Timms KM, Reid J, Homologous recombination deficiency (HRD) score predicts response to platinum-containing neoadjuvant chemotherapy in patients with triple-negative breast cancer. Clin Cancer Res 2016;22:3764–73. 26957554 10.1158/1078-0432.CCR-15-2477 PMC6773427

[60] Staaf J, Glodzik D, Bosch A, Whole-genome sequencing of triple-negative breast cancers in a population-based clinical study. Nat Med 2019;25:1526–33. 31570822 10.1038/s41591-019-0582-4 PMC6859071

[61] Chopra N, Tovey H, Pearson A, Homologous recombination DNA repair deficiency and PARP inhibition activity in primary triple negative breast cancer. Nat Commun 2020;11:2662. 32471999 10.1038/s41467-020-16142-7 PMC7260192

[62] Gulhan DC, Lee JJ, Melloni GEM, Detecting the mutational signature of homologous recombination deficiency in clinical samples. Nat Genet 2019;51:912–9. 30988514 10.1038/s41588-019-0390-2

[63] Quereda V, Bayle S, Vena F, Therapeutic targeting of CDK12/CDK13 in triple-negative breast cancer. Cancer Cell 2019;36:545–58.e7. 31668947 10.1016/j.ccell.2019.09.004

[64] Cantley LC. The phosphoinositide 3-kinase pathway. Science 2002;296:1655–7. 12040186 10.1126/science.296.5573.1655

[65] Genome Atlas Network. Comprehensive molecular portraits of human breast tumours. Nature 2012;490:61–70. 23000897 10.1038/nature11412 PMC3465532

[66] Curtis C, Shah SP, Chin SF, The genomic and transcriptomic architecture of 2,000 breast tumours reveals novel subgroups. Nature 2012;486:346–52. 22522925 10.1038/nature10983 PMC3440846

[67] Schmid P, Abraham J, Chan S, Capivasertib plus paclitaxel versus placebo plus paclitaxel as first-line therapy for metastatic triple-negative breast cancer: The PAKT trial. J Clin Oncol 2020;38:423–33. 31841354 10.1200/JCO.19.00368

[68] Takeshita T, Yamamoto Y, Yamamoto-Ibusuki M, Clinical significance of plasma cell-free DNA mutations in PIK3CA, AKT1, and ESR1 gene according to treatment lines in ER-positive breast cancer. Mol Cancer 2018;17:67. 29482551 10.1186/s12943-018-0808-y PMC6389169

[69] Yuan H, Chen J, Liu Y, Association of PIK3CA mutation status before and after neoadjuvant chemotherapy with response to chemotherapy in women with breast cancer. Clinical Cancer Research 2015;21:4365–72. 25979484 10.1158/1078-0432.CCR-14-3354

[70] Bareche Y, Venet D, Ignatiadis M, Unravelling triple-negative breast cancer molecular heterogeneity using an integrative multiomic analysis. Ann Oncol 2018;29:895–902. 29365031 10.1093/annonc/mdy024 PMC5913636

[71] Kim SB, Dent R, Im SA, Ipatasertib plus paclitaxel versus placebo plus paclitaxel as first-line therapy for metastatic triple-negative breast cancer (LOTUS): a multicentre, randomised, double-blind, placebo-controlled, phase 2 trial. Lancet Oncol 2017;18:1360–72. 28800861 10.1016/S1470-2045(17)30450-3 PMC5626630

[72] Dent R, Kim SB, Oliveira M, Double-blind placebo (PBO)-controlled randomized phase III trial evaluating first-line ipatasertib (IPAT) combined with paclitaxel (PAC) for PIK3CA/AKT1/PTEN-altered locally advanced unresectable or metastatic triple-negative breast cancer (aTNBC): primary results from IPATunity130 Cohort A. Proceedings of the 2020 Virtual San Antonio Breast Cancer Symposium; 2020 Dec 8–11; San Antonio, TX. Philadelphia (PA): AACR; 2021.

[73] Verma S, Miles D, Gianni L, Trastuzumab emtansine for HER2-positive advanced breast cancer. N Engl J Med 2012;367:1783–91. 23020162 10.1056/NEJMoa1209124 PMC5125250

[74] von Minckwitz G, Huang CS, Mano MS, Trastuzumab emtansine for residual invasive HER2-positive breast cancer. N Engl J Med 2019;380:617–28. 30516102 10.1056/NEJMoa1814017

[75] Modi S, Saura C, Yamashita T, Trastuzumab Deruxtecan in previously treated HER2-positive breast cancer. N Engl J Med 2020;382:610–21. 31825192 10.1056/NEJMoa1914510 PMC7458671

[76] Bardia A, Mayer IA, Vahdat LT, Sacituzumab Govitecan-hziy in refractory metastatic triple-negative breast cancer. N Engl J Med 2019;380:741–51. 30786188 10.1056/NEJMoa1814213

[77] Sacituzumab govitecan package insert. Available from : https://www.accessdata.fda.gov/drugsatfda_docs/label/2020/761115s000lbl.pdf. [Last accessed on 24 Mar 2021].

[78] Bardia A, Tolaney SM, Loirat D, ASCENT: A randomized phase III study of sacituzumab govitecan (SG) vs treatment of physician’s choice (TPC) in patients (pts) with previously treated metastatic triple-negative breast cancer (mTNBC). Ann Oncol 2020;31:S1149–50.

[79] Hurvitz SA, Tolaney SM, Punie K, Biomarker evaluation in the phase 3 ASCENT study of sacituzumab govitecan versus chemotherapy in patients with metastatic triple-negative breast cancer. San Antonio Breast Cancer Symposium 2020.

[80] Han H, Diab S, Alemany C, Open label phase 1b/2 study of ladiratuzumab vedotin in combination with pembrolizumab for first-line treatment of patients with unresectable locally-advanced or metastatic triple-negative breast cancer. Proceedings of the 2019 San Antonio Breast Cancer Symposium; 2019 Dec 10–14; San Antonio, USA. 2020.

[81] Ogitani Y, Hagihara K, Oitate M, Bystander killing effect of DS-8201a, a novel anti-human epidermal growth factor receptor 2 antibody-drug conjugate, in tumors with human epidermal growth factor receptor 2 heterogeneity. Cancer Sci 2016;107:1039–46. 27166974 10.1111/cas.12966 PMC4946713

[82] Ogitani Y, Aida T, Hagihara K, DS-8201a, A novel HER2-targeting ADC with a novel DNA topoisomerase I inhibitor, demonstrates a promising antitumor efficacy with differentiation from T-DM1. Clin Cancer Res 2016;22:5097–108. 27026201 10.1158/1078-0432.CCR-15-2822

[83] Modi S, Park H, Murthy RK, Antitumor activity and safety of Trastuzumab Deruxtecan in patients with HER2-low-expressing advanced breast cancer: Results from a phase Ib study. J Clin Oncol 2020;38:1887–96. 32058843 10.1200/JCO.19.02318 PMC7280051

[84] Banerji U, van Herpen CML, Saura C, Trastuzumab duocarmazine in locally advanced and metastatic solid tumors and HER2-expressing breast cancer: A phase 1 dose-escalation and dose-expansion study. Lancet Oncol 2019;20:1124–35. 31257177 10.1016/S1470-2045(19)30328-6

[85] Tolcher AW. The evolution of antibody-drug conjugates: A positive inflexion point. Am Soc Clin Oncol Educ Book 2020;40:1–8. 10.1200/EDBK_281103 32223669

[86] Doane AS, Danso M, Lal P, An estrogen receptor-negative breast cancer subset characterized by a hormonally regulated transcriptional program and response to androgen. Oncogene 2006;25:3994–4008. 16491124 10.1038/sj.onc.1209415

[87] Loibl S, Müller BM, von Minckwitz G, Androgen receptor expression in primary breast cancer and its predictive and prognostic value in patients treated with neoadjuvant chemotherapy. Breast Cancer Res Treat 2011;130:477–87. 21837479 10.1007/s10549-011-1715-8

[88] Cochrane DR, Bernales S, Jacobsen BM, Role of the androgen receptor in breast cancer and preclinical analysis of enzalutamide. Breast Cancer Res 2014;16:R7. 24451109 10.1186/bcr3599 PMC3978822

[89] Jia LY, Shanmugam MK, Sethi G, Potential role of targeted therapies in the treatment of triple-negative breast cancer. Anticancer Drugs 2016;27:147–55. 26682525 10.1097/CAD.0000000000000328

[90] Gucalp A, Tolaney S, Isakoff SJ, Phase II trial of bicalutamide in patients with androgen receptor-positive, estrogen receptor-negative metastatic breast cancer. Clin Cancer Res 2013;19:5505–12. 23965901 10.1158/1078-0432.CCR-12-3327 PMC4086643

[91] Bonnefoi H, Grellety T, Tredan O, A phase II trial of abiraterone acetate plus prednisone in patients with triple-negative androgen receptor positive locally advanced or metastatic breast cancer (UCBG 12–1). Ann Oncol 2016;27:812–8. 27052658 10.1093/annonc/mdw067

[92] Traina TA, Miller K, Yardley DA, Enzalutamide for the treatment of androgen receptor-expressing triple-negative breast cancer. J Clin Oncol 2018;36:884–90. 29373071 10.1200/JCO.2016.71.3495 PMC5858523

[93] Schumacher TN, Schreiber RD. Neoantigens in cancer immunotherapy. Science 2015;348:69–74. 25838375 10.1126/science.aaa4971

[94] Ahn J, Xia T, Rabasa Capote A, Extrinsic phagocyte-dependent STING signaling dictates the immunogenicity of dying cells. Cancer Cell 2018;33:862–73.e5. 29706455 10.1016/j.ccell.2018.03.027 PMC6177226

[95] Sharma P, Allison JP. The future of immune checkpoint therapy. Science 2015;348:56–61. 25838373 10.1126/science.aaa8172

[96] Loi S, Drubay D, Adams S, Tumor-infiltrating lymphocytes and prognosis: A pooled individual patient analysis of early-stage triple-negative breast cancers. J Clin Oncol 2019;37:559–69. 30650045 10.1200/JCO.18.01010 PMC7010425

[97] Topalian SL, Drake CG, Pardoll DM. Immune checkpoint blockade: a common denominator approach to cancer therapy. Cancer Cell 2015;27:450–61. 25858804 10.1016/j.ccell.2015.03.001 PMC4400238

[98] Karn T, Jiang T, Hatzis C, Association between genomic metrics and immune infiltration in triple-negative breast cancer. JAMA Oncol 2017;3:1707–11. 28750120 10.1001/jamaoncol.2017.2140 PMC5824276

[99] Dushyanthen S, Beavis PA, Savas P, Relevance of tumor-infiltrating lymphocytes in breast cancer. BMC Med 2015;13:202. 26300242 10.1186/s12916-015-0431-3 PMC4547422

[100] Azizi E, Carr AJ, Plitas G, Single-cell map of diverse immune phenotypes in the breast tumor microenvironment. Cell 2018;174:1293–308.e36. 29961579 10.1016/j.cell.2018.05.060 PMC6348010

[101] Chung W, Eum HH, Lee HO, Single-cell RNA-seq enables comprehensive tumour and immune cell profiling in primary breast cancer. Nat Commun 2017;8:15081. 28474673 10.1038/ncomms15081 PMC5424158

[102] Wagner J, Rapsomaniki MA, Chevrier S, A single-cell atlas of the tumor and immune ecosystem of human breast cancer. Cell 2019;177:1330–45.e18. 30982598 10.1016/j.cell.2019.03.005 PMC6526772

[103] Kim IS, Gao Y, Welte T, Immuno-subtyping of breast cancer reveals distinct myeloid cell profiles and immunotherapy resistance mechanisms. Nat Cell Biol 2019;21:1113–26. 31451770 10.1038/s41556-019-0373-7 PMC6726554

[104] Naik A, Decock J. Lactate metabolism and immune modulation in breast cancer: A focused review on triple negative breast tumors. Front Oncol 2020;10:598626. 33324565 10.3389/fonc.2020.598626 PMC7725706

[105] Pilon-Thomas S, Kodumudi KN, El-Kenawi AE, Neutralization of tumor acidity improves antitumor responses to immunotherapy. Cancer Res 2016;76:1381–90. 26719539 10.1158/0008-5472.CAN-15-1743 PMC4829106

[106] Pötzl J, Roser D, Bankel L, Reversal of tumor acidosis by systemic buffering reactivates NK cells to express IFN-γ and induces NK cell-dependent lymphoma control without other immunotherapies. Int J Cancer 2017;140:2125–33. 28195314 10.1002/ijc.30646

[107] O’Sullivan D, Sanin DE, Pearce EJ, Metabolic interventions in the immune response to cancer. Nat Rev Immunol 2019;19:324–35. 30820043 10.1038/s41577-019-0140-9

[108] Nanda R, Chow LQ, Dees EC, Pembrolizumab in patients with advanced triple-negative breast cancer: phase Ib KEYNOTE-012 study. J Clin Oncol 2016;34:2460–7. 27138582 10.1200/JCO.2015.64.8931 PMC6816000

[109] Adams S, Schmid P, Rugo HS, Pembrolizumab monotherapy for previously treated metastatic triple-negative breast cancer: cohort A of the phase II KEYNOTE-086 study. Ann Oncol 2019;30:397–404. 30475950 10.1093/annonc/mdy517

[110] Szekely B, Bossuyt V, Li X, Immunological differences between primary and metastatic breast cancer. Ann Oncol 2018;29:2232–9. 30203045 10.1093/annonc/mdy399

[111] Hutchinson KE, Yost SE, Chang CW, Comprehensive profiling of poor-risk paired primary and recurrent triple-negative breast cancers reveals immune phenotype shifts. Clin Cancer Res 2020;26:657–68. 31611282 10.1158/1078-0432.CCR-19-1773 PMC8568263

[112] Nanda R, Liu MC, Yau C, Shatsky R, Effect of pembrolizumab plus neoadjuvant chemotherapy on pathologic complete response in women with early-stage breast cancer: An analysis of the ongoing phase 2 adaptively randomized I-SPY2 trial. JAMA Oncol 2020;6:676–84. 32053137 10.1001/jamaoncol.2019.6650 PMC7058271

[113] Schmid P, Cortes J, Pusztai L, Pembrolizumab for early triple-negative breast cancer. N Engl J Med 2020;382:810–21. 32101663 10.1056/NEJMoa1910549

[114] Oncologic Drugs Advisory Committee (ODAC) Meeting Briefing Materials. Available from: https://www.fda.gov/media/145654/download. [Last accessed on 24 Mar 2021].

[115] Mittendorf EA, Zhang H, Barrios CH, Neoadjuvant atezolizumab in combination with sequential nab-paclitaxel and anthracycline-based chemotherapy versus placebo and chemotherapy in patients with early-stage triple-negative breast cancer (IMpassion031): a randomised, double-blind, phase 3 trial. Lancet 2020;396:1090–100. 32966830 10.1016/S0140-6736(20)31953-X

[116] Mittendorf EA, Harbeck N, Zhang H, Patient-reported outcomes (PROs) from the Ph 3 IMpassion031 trial of neoadjuvant (NA) atezolizumab + chemo in early triple-negative breast cancer (eTNBC). Proceedings of the 2020 Virtual San Antonio Breast Cancer Symposium. 2020 Dec 8–11; San Antonio, TX. Philadelphia (PA): AACR; 2021.

[117] Gianni L, Huang CS, Egle D, Pathologic complete response (pCR) to neoadjuvant treatment with or without atezolizumab in triple negative, early high-risk and locally advanced breast cancer. NeoTRIPaPDL1 Michelangelo randomized study. Proceedings of the 2019 San Antonio Breast Cancer Symposium. 2019 Dec 10–14; San Antonio, TX. Philadelphia (PA): AACR; 2020. 10.1016/j.annonc.2022.02.004 35182721

[118] Voorwerk L, Slagter M, Horlings HM, Immune induction strategies in metastatic triple-negative breast cancer to enhance the sensitivity to PD-1 blockade: the TONIC trial. Nat Med 2019;25:920–8. 31086347 10.1038/s41591-019-0432-4

[119] Loibl S, Untch M, Burchardi N, A randomised phase II study investigating durvalumab in addition to an anthracycline taxane-based neoadjuvant therapy in early triple-negative breast cancer: clinical results and biomarker analysis of GeparNuevo study. Ann Oncol 2019;30:1279–88. 31095287 10.1093/annonc/mdz158

[120] Cortés J, Lipatov O, Im S, KEYNOTE-119: phase 3 study of pembrolizumab (pembro) versus single-agent chemotherapy (chemo) for metastatic triple negative breast cancer (MTNBC). Ann Oncol 2019;30:v851–934.

[121] Dirix LY, Takacs I, Jerusalem G, Avelumab, an anti-PD-L1 antibody, in patients with locally advanced or metastatic breast cancer: a phase 1b JAVELIN solid tumor study. Breast Cancer Res 2018;167:671–86. 10.1007/s10549-017-4537-5 PMC5807460 29063313

[122] Galluzzi L, Buqué A, Kepp O, Immunological effects of conventional chemotherapy and targeted anticancer agents. Cancer Cell 2015;28:690–714. 26678337 10.1016/j.ccell.2015.10.012

[123] Schmid P, Adams S, Rugo HS, Atezolizumab and nab-paclitaxel in advanced triple-negative breast cancer. N Engl J Med 2018;379:2108–21. 30345906 10.1056/NEJMoa1809615

[124] Emens LA, Adams S, Barrios CH, IMpassion130: Final OS analysis from the pivotal phase III study of atezolizumab + nab-paclitaxel vs placebo + nab-paclitaxel in previously untreated locally advanced or metastatic triple-negative breast cancer. Ann Oncol 2020;31:S1148.

[125] Miles DW, Gligorov J, André F, Primary results from IMpassion131, a double-blind placebo-controlled randomised phase III trial of first-line paclitaxel (PAC) ± atezolizumab (atezo) for unresectable locally advanced/metastatic triple-negative breast cancer (mTNBC). Ann Oncol 2020;31:S1147–8. 10.1016/j.annonc.2021.05.801 34219000

[126] Rugo HS, Schmid P, Cescon DW, Additional efficacy endpoints from the phase 3 KEYNOTE-355 study of pembrolizumab plus chemotherapy vs placebo plus chemotherapy as first-line therapy for locally recurrent inoperable or metastatic triple-negative breast cancer. San Antonio Breast Cancer Symposium 2020.

[127] Cortés J, Cescon DW, Rugo HS, KEYNOTE-355: Randomized, double-blind, phase III study of pembrolizumab + chemotherapy versus placebo + chemotherapy for previously untreated locally recurrent inoperable or metastatic triple-negative breast cancer. J Clin Oncol 2020;38:1000. 10.1016/S0140-6736(20)32531-9 33278935

[128] Huang X, Ding Q, Guo H, Comparison of three FDA-approved diagnostic immunohistochemistry assays of PD-L1 in triple-negative breast carcinoma. Hum Pathol 2020;108:42–50. 33221342 10.1016/j.humpath.2020.11.004

[129] Lee SE, Park HY, Lim SD, Concordance of Programmed death-ligand 1 expression between SP142 and 22C3/SP263 assays in triple-negative breast cancer. J Breast Cancer 2020;23:303–13. 32595992 10.4048/jbc.2020.23.e37 PMC7311361

[130] Noske A, Ammann JU, Wagner DC, A multicentre analytical comparison study of inter-reader and inter-assay agreement of four programmed death-ligand 1 immunohistochemistry assays for scoring in triple-negative breast cancer. Histopathology 2021;78:567–77. 32936950 10.1111/his.14254

[131] Gonzalez-Ericsson PI, Stovgaard ES, Sua LF, The path to a better biomarker: application of a risk management framework for the implementation of PD-L1 and TILs as immuno-oncology biomarkers in breast cancer clinical trials and daily practice. J Pathol 2020;250:667–84. 32129476 10.1002/path.5406

[132] Samstein RM, Lee CH, Shoushtari AN, Tumor mutational load predicts survival after immunotherapy across multiple cancer types. Nat Genet 2019;51:202–6. 30643254 10.1038/s41588-018-0312-8 PMC6365097

[133] Barroso-Sousa R, Keenan TE, Pernas S, Tumor mutational burden and PTEN alterations as molecular correlates of response to PD-1/L1 blockade in metastatic triple-negative breast cancer. Clin Cancer Res 2020;26:2565–72. 32019858 10.1158/1078-0432.CCR-19-3507 PMC7269810

[134] Lu S, Stein JE, Rimm DL, Comparison of biomarker modalities for predicting response to PD-1/PD-L1 checkpoint blockade: A systematic review and meta-analysis. JAMA Oncol 2019;5:1195–204. 31318407 10.1001/jamaoncol.2019.1549 PMC6646995

[135] Keren L, Bosse M, Marquez D, A structured tumor-immune microenvironment in triple negative breast cancer revealed by multiplexed Ion beam imaging. Cell 2018;174:1373–87.e19. 30193111 10.1016/j.cell.2018.08.039 PMC6132072

[136] Schürch CM, Bhate SS, Barlow GL, Coordinated cellular neighborhoods orchestrate antitumoral immunity at the colorectal cancer invasive front. 2020;182:1341–59.e19. 10.1016/j.cell.2020.07.005 PMC7479520 32763154

[137] Coombes RC, Page K, Salari R, Personalized detection of circulating tumor DNA antedates breast cancer metastatic recurrence. Clin Cancer Res 2019;25:4255–63. 30992300 10.1158/1078-0432.CCR-18-3663

[138] Bratman SV, Yang SY, Iafolla MAJ, Personalized circulating tumor DNA analysis as a predictive biomarker in solid tumor patients treated with pembrolizumab. Nat Cancer 2020;1:873–81. 10.1038/s43018-020-0096-5 35121950

[139] Volovat SR, Volovat C, Hordila I, MiRNA and LncRNA as potential biomarkers in triple-negative breast cancer: A review. Front Oncol 2020;10:526850. 33330019 10.3389/fonc.2020.526850 PMC7716774

[140] Piña-Sánchez P, Valdez-Salazar H, Ruiz-Tachiquín M. Circulating microRNAs and their role in the immune response in triple-negative breast cancer. Oncol Lett 2020;20:224. 32968446 10.3892/ol.2020.12087 PMC7499949

[141] Yu Y, Zhang W, Li A, Association of long noncoding RNA biomarkers with clinical immune subtype and prediction of immunotherapy response in patients with cancer. JAMA Netw Ope 2020;3:e202149. 10.1001/jamanetworkopen.2020.2149 PMC7139278 32259264

